# KRAS-specific inhibition using a DARPin binding to a site in the allosteric lobe

**DOI:** 10.1038/s41467-019-10419-2

**Published:** 2019-06-13

**Authors:** Nicolas Bery, Sandrine Legg, Judit Debreczeni, Jason Breed, Kevin Embrey, Christopher Stubbs, Paulina Kolasinska-Zwierz, Nathalie Barrett, Rose Marwood, Jo Watson, Jon Tart, Ross Overman, Ami Miller, Christopher Phillips, Ralph Minter, Terence H. Rabbitts

**Affiliations:** 1 Weatherall Institute of Molecular Medicine, MRC Molecular Haematology Unit, University of Oxford, John Radcliffe Hospital, Oxford, OX3 9DS UK; 2 Antibody Discovery and Protein Engineering, R&D BioPharmaceuticals, Milstein Building Granta Park, Cambridge, CB21 6GH UK; 3 Discovery Sciences, R&D BioPharmaceuticals, AstraZeneca, Darwin Building, Cambridge Science Park, Milton Road, Cambridge, CB4 0WG UK

**Keywords:** X-ray crystallography, Cancer, Drug discovery, Molecular biology

## Abstract

Inhibiting the RAS oncogenic protein has largely been through targeting the switch regions that interact with signalling effector proteins. Here, we report designed ankyrin repeat proteins (DARPins) macromolecules that specifically inhibit the KRAS isoform by binding to an allosteric site encompassing the region around KRAS-specific residue histidine 95 at the helix α3/loop 7/helix α4 interface. We show that these DARPins specifically inhibit KRAS/effector interactions and the dependent downstream signalling pathways in cancer cells. Binding by the DARPins at that region influences KRAS/effector interactions in different ways, including KRAS nucleotide exchange and inhibiting KRAS dimerization at the plasma membrane. These results highlight the importance of targeting the α3/loop 7/α4 interface, a previously untargeted site in RAS, for specifically inhibiting KRAS function.

## Abstract (web summary)

Mutant RAS family members occur in a wide range of tumour types, and there is a great interest in identifying isoform-specific inhibitors. Here, the authors characterise two designed ankyrin repeat proteins (DARPins) that specifically inhibit the KRAS isoform by binding to the region around the KRAS-specific residue histidine 95 and show that they affect KRAS/effector interactions in different ways.

## Introduction

Members of the RAS family of oncogenic proteins are frequently mutated in human cancers. In particular, *KRAS* mutations are the most prominent ones, representing around 86% of all RAS mutations^1^. KRAS mutants are major drivers of cancers, such as colorectal, lung or pancreatic cancers^1^. Isolation of selective KRAS inhibitors that block its function is therefore an important goal^2^. Nonetheless, selectively targeting KRAS is challenging, as RAS isoforms are highly similar in primary sequence with 82–90% amino acid sequence identity^3^.

Most current inhibitors target all RAS isoforms via their conserved effector lobe (defined as amino acid 1–86) by inhibiting RAS/effector interactions^4–7^ or RAS nucleotide exchange^8,9^. We identified such pan-RAS inhibitors in a previous study with the anti-RAS designed ankyrin repeat proteins (DARPins) K55 (RAS/effector interactions inhibitor) and K27 (RAS nucleotide exchange inhibitor)^8^. As an alternative, targeting RAS via its allosteric lobe (amino acids 87–166)^10^ is a possible way to inhibit its function in cells^11–13^. The α3–α4 and α4–α5 interface in the allosteric lobe are potential dimerisation sites for RAS^14–17^ and preventing KRAS dimerisation impairs the mitogen-activated protein kinase (MAPK) signalling pathway^18^. Recent studies have shown that dimerisation is a potential targetable feature of KRAS function^11–13^. Notably, a monobody that targets both HRAS and KRAS on the α4–α5 site, disrupts RAS dimerisation, blocks RAF activation^12^ and inhibits tumour formation in vivo^13^. Nevertheless, none of these inhibitors are KRAS selective.

Specifically targeting directly mutant KRAS has been achieved with small molecules covalently binding the G12C mutant KRAS^19–21^. This approach targets the G12C mutation that represents around 12% of KRAS mutations in cancers (Cosmic database v86, https://cosmic-blog.sanger.ac.uk/), and is only present in a subset of cancers, such as non-small cell lung cancers^22^. Therefore, alternative strategies are needed to inhibit the most frequent mutations of KRAS accounting for 88% of KRAS mutant cancers.

We report here the characterisation of two potent DARPins that selectively bind KRAS on a site of the allosteric lobe, encompassing histidine residue 95. The DARPin binding inhibits KRAS nucleotide exchange and KRAS dimerisation, thus impairing mutant KRAS–effector interactions and the downstream signalling pathways. These findings reveal a unique strategy to selectively inhibit KRAS.

## Results

### Isolation of anti-KRAS-specific DARPins

We performed a phage display selection of a diverse DARPin library^8^, followed by immunoassays with KRAS^G12V^ to isolate hits. We have identified two DARPins (designated K13 and K19) that bound to KRAS^G12V^. Biochemical analysis of the DARPins show K13 and K19 interact with KRAS independently of the nucleotide-bound state of the GTPase, and have K_d_s around 30 and 10 nM, respectively (Supplementary Fig. 1a). The nucleotide and protein sequences of DARPins K13 and K19 are shown in Supplementary Fig. 1b, c and highlight a conserved amino acid sequence in the repeat regions with only six amino acids difference.

**Fig. 1:**
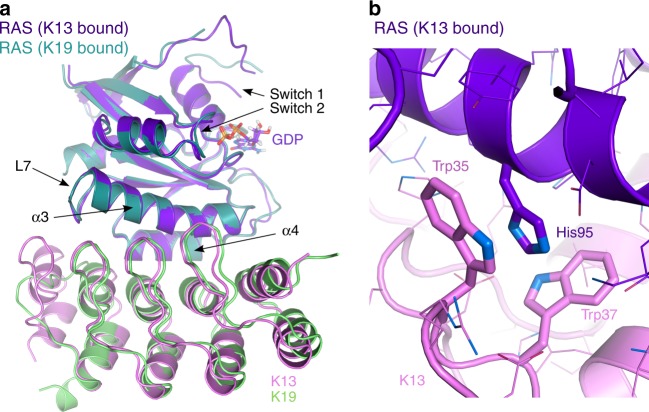
DARPins K13 and K19 bind KRAS to a novel allosteric site. **a** X-ray crystal structures of KRAS^G12V^-GDP-bound to DARPin K13 (PDB 6H46) and KRAS^G12V^-GDP-bound to DARPin K19 (PDB 6H47) with the indication of RAS switch regions, helix 3 (α3), helix 4 (α4) and loop 7 (L7) regions. **b** Expanded view of the interaction between the DARPin K13 and KRAS around histidine residue 95 of KRAS

**Fig. 2:**
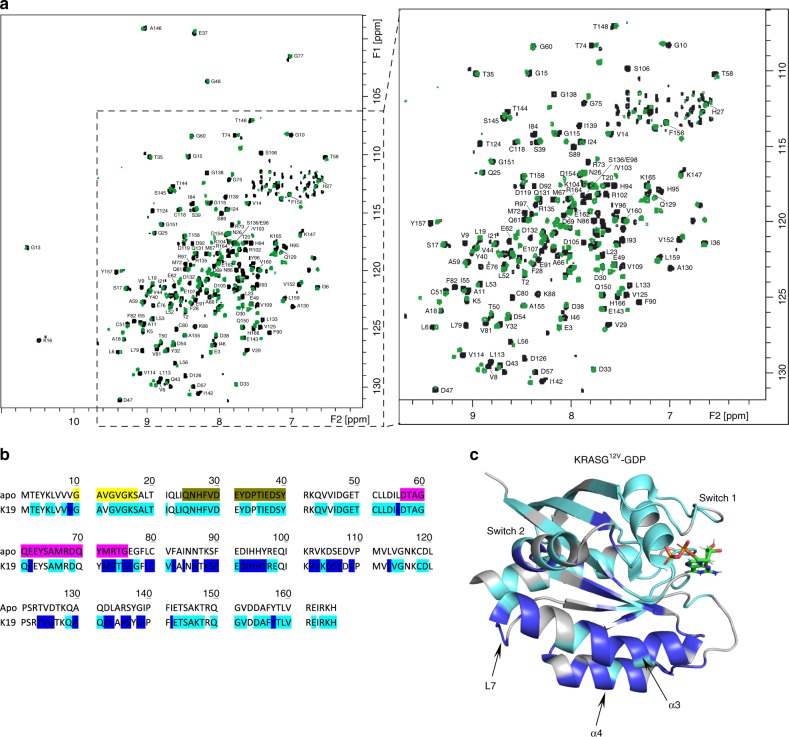
HSQC NMR chemical shift changes in KRAS after DARPin K19 binding. **a** Comparison between ^1^H, ^15^N TROSY spectra for apo KRAS^G12V^ at 100 μM (black) overlaid with K19-bound KRAS^G12V^ at 89 μM (green). DARPin concentrations were in slight excess. An expanded region is shown on the right. **b** Amino acids undergoing chemical shifts upon DARPin K19 binding on KRAS^G12V^-GDP are shown in blue, residues not experiencing shifts are shown in turquoise in the KRAS^G12V^ sequence (unassigned residues are not highlighted). The P-loop is highlighted in yellow, the switch 1 in dark yellow and the switch 2 in purple. **c** Ribbon representation of (**b**) with amino acids undergoing chemical shifts in blue and residues not experiencing shifts in turquoise in the KRAS^G12V^-GDP structure (PDB 4TQ9) after DARPin K19 binding (unassigned residues are in grey). L7: loop 7, α3: helix 3, α4: helix 4

The X-ray structure data of K13 and K19 in complex with KRAS^G12V^ show these DARPins bind to the allosteric lobe of KRAS, at the interface between helix α3/loop 7/helix α4 (Fig. 1a, b; Supplementary Table 1). The crystal structures show that when DARPins K13 or K19 bind to KRAS, a structural change appears in the KRAS molecule on the effector lobe, especially on the switch 1 and 2 when compared with two unbound KRAS^G12V^-GDP structures (Supplementary Fig. 2a, b). However, the exact conformation of the switch 1 loop in the K13- and K19-bound states differ somewhat. This difference is most likely due to their different crystal-packing environments (Supplementary Fig. 2c, d). NMR chemical shift perturbation HSQC and hydrogen deuterium exchange with mass spectrometry (HDX-MS) data support the observed binding interface in solution of K19 in the allosteric lobe (Fig. 2a–c and Supplementary Figs. 3–5) and control DARPin K27 in the effector lobe (previously shown to interact with the switch regions of KRAS, NRAS and HRAS-GDP^8^) (Supplementary Figs. 4–6). After K19 binding to KRAS, a small but significant increase in the dynamic mobility of the switch 2 loop is shown by the increase in de-protection observed by HDX-MS (Supplementary Figs. 3–4), and some small perturbations of the effector lobe HSQC resonances are observed in a few residues in the switch 2 region (Fig. 2a–c). Our data suggest that the conformational change observed by X-ray crystallography on the switch regions is most likely due to the crystal-packing effect, because the switch regions are flexible by nature.

### DARPins K13 and K19 are KRAS selective binders

**Fig. 3:**
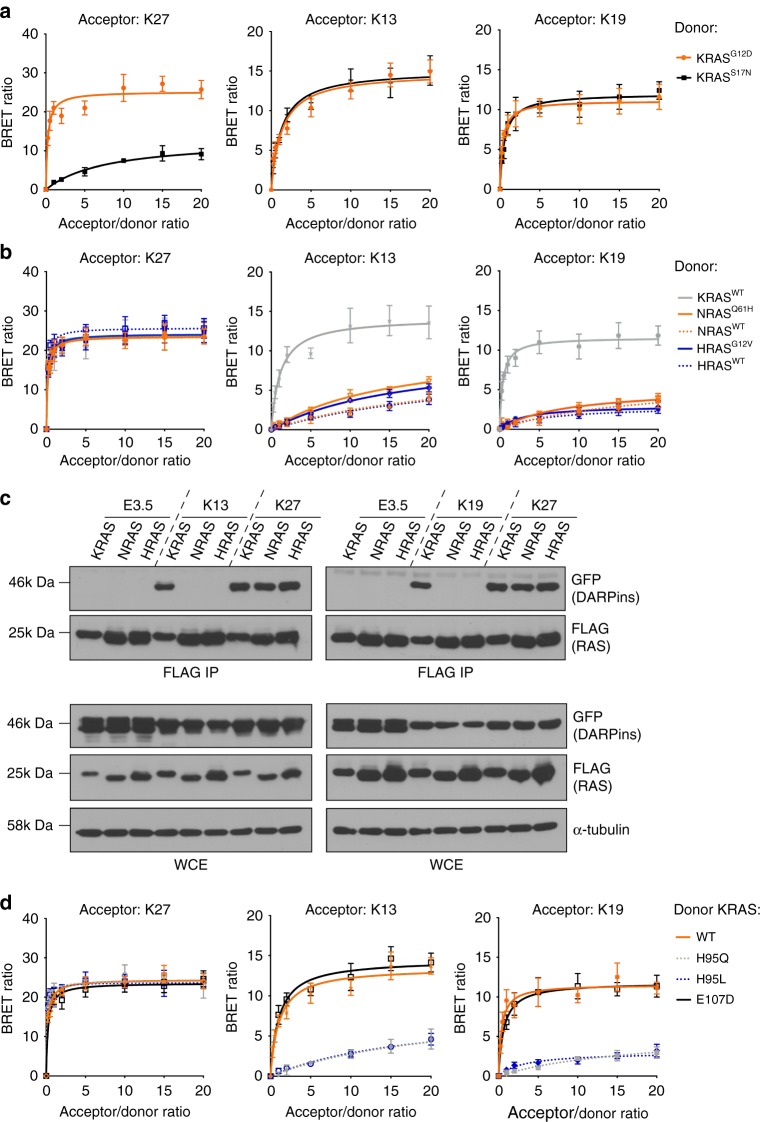
K13 and K19 are KRAS selective binders. **a** BRET donor saturation assay between KRAS^G12D^ or KRAS^S17N^ (donors) and the DARPins (acceptors). **b** BRET donor saturation assay between KRAS^WT^, NRAS^WT^, NRAS^Q61H^, HRAS^WT^ or HRAS^G12V^ (donors) and the DARPins (acceptors). **c** Co-immunoprecipitation of 3xFLAG-KRAS^WT^, −NRAS^WT^ and −HRAS^WT^ with the DARPin-GFP^2^ fusions in 10% foetal bovine serum. IP   Immunoprecipitation, WCE   whole-cell extract. **d** BRET donor saturation assay between KRAS^WT^, KRAS^H95Q^, KRAS^H95L^ or KRAS^E107D^ (donors) and the DARPins (acceptors). Note that most of the donor saturation curves with K27 as acceptor are overlapping in panels **b** and **d**. Each experiment was performed three times (**a**–**d**). Error bars are mean ± SD of biological repeats. **c** Source data are provided as a Source Data file

K13 and K19 interact with the allosteric lobe where the amino acids between the RAS isoforms are less conserved than the effector lobe. Thus, we assessed whether K13 and K19 could bind to all RAS family isoforms. While the switch region-binding DARPin K27 binds to K, N and HRAS in cell-based bioluminescent resonance energy transfer 2 (BRET2) assay^23^, K13 and K19 show only strong binding with KRAS (Fig. 3a, b). In addition, K13 and K19 interact equally with the dominant-negative KRAS^S17N^, the constitutively activated mutant KRAS^G12D^ and wild-type KRAS, unlike K27, which does not contact the KRAS^S17N^ mutant (Fig. 3a, b). Indeed, K13 and K19 bind KRAS away from the switch regions, and are not affected by the S17N mutation while K27 binding is expected to be affected as the flexibility of the switch regions is modified on that mutant^24^. These selectivity findings were further supported using a co-immunoprecipitation assay with cells co-expressing 3xFLAG-tagged wild-type RAS and GFP-tagged DARPin. KRAS, NRAS and HRAS proteins were immunoprecipitated on beads, and the presence of DARPin–GFP bound to RAS proteins was assessed. The DARPin K27 was captured by all three RAS isoforms, but K13 and K19 were only captured by KRAS, while the negative control DARPin E3.5^8^ was not co-immunoprecipitated by any RAS proteins (Fig. 3c).

### K13 and K19 bind KRAS by interacting with the H95 of KRAS

We evaluated which residue(s) are involved in this KRAS selectivity of K13 and K19. Analysis of the amino acids engaged in the interaction between the DARPins and KRAS, showed that K13 and K19 make extensive interactions with the histidine 95 and the glutamic acid 107, which are residues only present in KRAS (Supplementary Fig. 7a). Therefore, we introduced mutations on the positions 95 and 107 of wild-type (WT) KRAS^WT^ substituting HIS95 with leucine or glutamine (found at residue 95 in NRAS and HRAS, respectively) and substituting GLU107 with aspartic acid (found at residue 107 in both NRAS and HRAS). While the E107D mutation did not affect K13, K19 or K27 DARPins binding with KRAS (Fig. 3d), H95Q and H95L mutations drastically decrease K13 and K19 binding on KRAS^WT^ but do not affect K27 binding as shown by BRET (Fig. 3d) or co-immunoprecipitation (Supplementary Fig. 7b). Supplementary Table 2 summarises the effects of RAS mutations on the binding of the DARPins. Finally, we assessed the residues of the DARPins important for binding to KRAS by mutating two tryptophans found in the first repeat of both K13 and K19 to glycine residues (Supplementary Fig. 1b, c) and analysing BRET interaction signal with KRAS^G12D^. These mutations induce a significant decrease of K13 and K19 binding to KRAS (Supplementary Fig. 7c, d). We conclude that the main driver for KRAS selectivity is the interaction between the H95 of KRAS with the W35 and W37 of the DARPins K13 and K19.

### DARPins K13 and K19 inhibit KRAS/effector interactions in cells

**Fig. 4:**
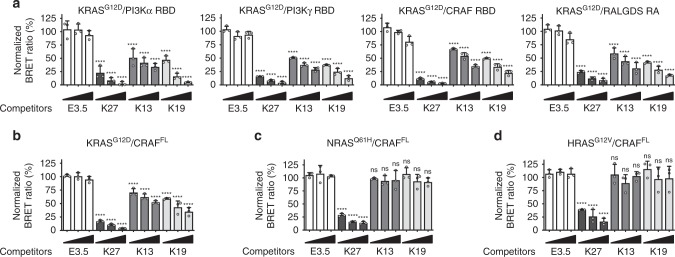
K13/K19 prevent KRAS/effector interactions in cells. **a** BRET competition assays between KRAS^G12D^/effector domain interactions and the DARPins used as competitors. The effector domains used were PI3Kα RBD, PI3Kγ RBD, CRAF RBD and RALGDS RAS-associating (RA) domain. **b**–**d** BRET competition assays between CRAF^FL^/KRAS^G12D^ (**b**), NRAS^Q61H^ (**c**) and HRAS^G12V^ (**d**) interactions with the DARPins used as competitors. E3.5 is a negative control, and DARPin K27 is a positive control. Statistical analyses in **a**–**d** were performed using a one-way ANOVA followed by Dunnett’s post-hoc tests (***P < 0.001, ****P < 0.0001, ns not significant). Each experiment was performed three times (**a**–**d**). Error bars are mean ± SD of biological repeats. **a**–**d** Source data are provided as a Source Data file

We assessed whether K13 and K19 interfere with KRAS function by inhibiting KRAS/effector interactions using the BRET assay. The DARPins were fused on their C terminus to a mCherry tag for monitoring their expression level (mCherry excitation and emission spectra do not overlap with those of our BRET2 donor or acceptor constructs)^25,26^. Unlike the negative control DARPin E3.5, K13 and K19 impede KRAS^G12D^ to interact with all the effector domains tested, including CRAF RBD (Fig. 4a; Supplementary Fig. 8a). Moreover, K13 and K19 selectively inhibit KRAS^G12D^/full-length CRAF^S257L^ (CRAF^FL^), but do not significantly alter NRAS^Q61H^/CRAF^FL^ or HRAS^G12V^/CRAF^FL^ interactions (Fig. 4b–d; Supplementary Fig. 8b), while K27 interferes with all three. Finally, K13 and K19 also induce a selective inhibition of the RAF/MEK/ERK signalling pathway mediated by KRAS^G12D^/CRAF^FL^ overexpression in HEK293T cells (Supplementary Fig. 9a, b). These results suggest that K13 and K19 are selective KRAS inhibitors.

### K13 and K19 inhibit KRAS-dependent signalling in cancer cells

**Fig. 5:**
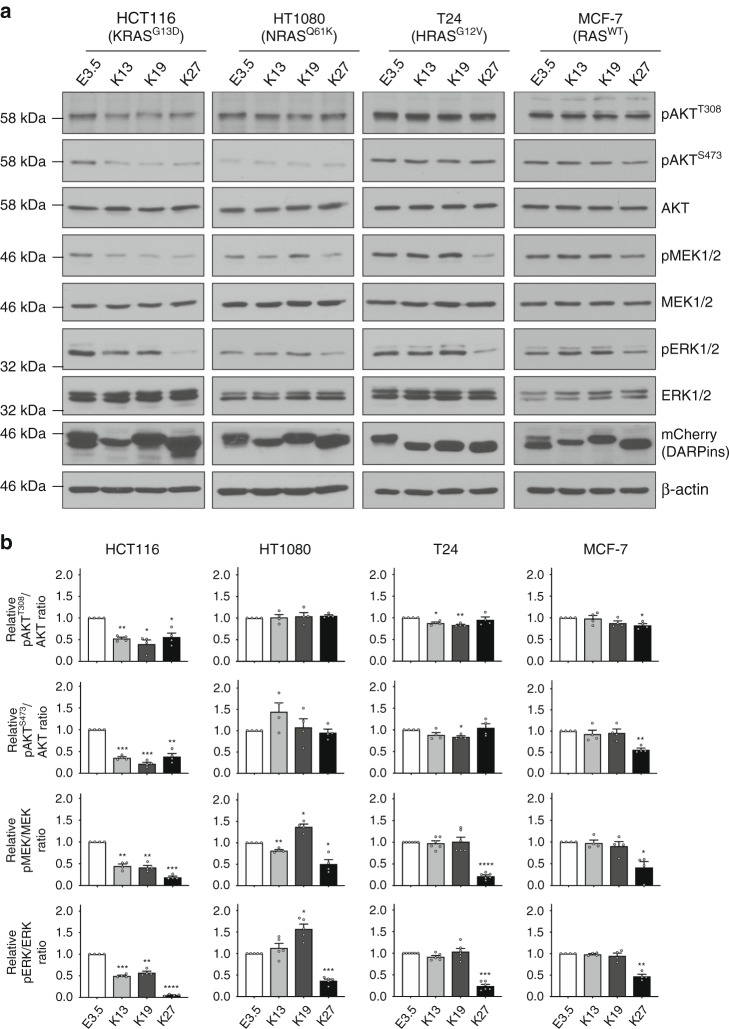
K13/K19 inhibitory potency in cells. **a** Western blot analyses of the activation state of the RAF/MEK/ERK and PI3K/AKT pathways in cancer cell lines bearing or not a mutated RAS upon overexpression of the DARPin-mCherry fusions. E3.5 is a negative and DARPin K27 is a positive control. **b** Quantifications of pERK/ERK, pMEK/MEK and pAKT/AKT signals from (**a**). These signals were normalised to the negative control DARPin E3.5. Statistical analyses in **b** were performed using a one-way ANOVA followed by Dunnett’s post-hoc tests (*P < 0.05, **P < 0.01, ***P < 0.001, ****P < 0.0001). Each experiment was performed at least four times (**a**–**b**). Error bars are mean ± SEM of biological repeats (**b**). **a**, **b** Source data are provided as a Source Data file

We tested the inhibitory effect of the DARPins in the following cancer cell lines: HCT116 (a colorectal cancer cell line, KRAS^G13D^), HT1080 (a fibrosarcoma cell line, NRAS^Q61K^), T24 (a bladder carcinoma cell line, HRAS^G12V^) and MCF-7 (a breast adenocarcinoma cell line, RAS^WT^). These were transiently transfected 24 h with the DARPins, and both RAF/MEK/ERK and PI3K/AKT pathways were assessed by western blot analysis. While K27 inhibits the RAF/MEK/ERK pathway in the three RAS mutant cell lines and the RAS^WT^ cancer cell line tested (consistent with its binding to the switch regions), K13 and K19 only decrease significantly the phosphorylation of MEK and ERK kinases in the KRAS mutant HCT116 cell line, but not in either the NRAS or the HRAS mutant cell lines or the RAS^WT^ cancer cell line (Fig. 5a, b). K13 and K19 DARPins selectively inhibit the PI3K/AKT pathway in the KRAS mutant HCT116 cell line, but not in the other cancer cell lines, while K27 inhibits this pathway in HCT116 and MCF-7 cells (Fig. 5a, b). These data are consistent with the finding that the PI3K/AKT pathway is only partly dependent on RAS, and can be activated independently of RAS^27,28^. These results demonstrate that K13 and K19 selectively inhibit endogenous mutant KRAS function without affecting RAS^WT^ cancer cells.

### K13 and K19 inhibit KRAS function by at least two mechanisms

**Fig. 6:**
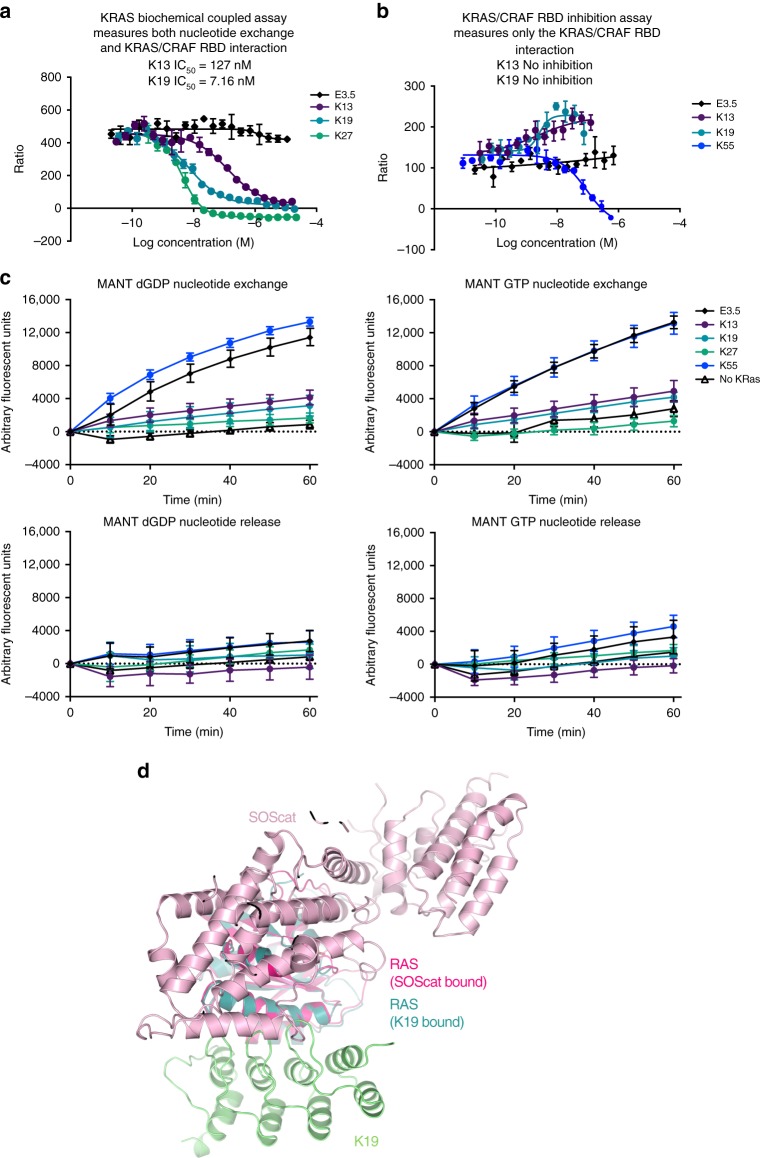
K13 and K19 inhibit KRAS nucleotide exchange. **a** KRAS biochemical coupled assay for inhibition of nucleotide exchange or KRAS/CRAF RBD interaction by DARPins K13, K19 or negative control DARPin E3.5. KRAS^G12V^ loaded with GDP was incubated for 15 min with a dilution series of DARPins K13, K19, K27 or E3.5. SOS and GTPγS were added to allow nucleotide exchange, followed by CRAF RBD. The KRAS^G12V^-GTPγS/CRAF RBD complex was quantitated by the FRET signal. **b** KRAS/CRAF RBD inhibition assay testing DARPins K13, K19, K55 (a known KRAS/CRAF RBD inhibitor) and E3.5. The FRET signal was measured for the interaction between CRAF RBD and KRAS^G12V^ loaded with GTPγS and inhibition of the signal monitored at varying concentrations of DARPins K13, K19, K55 and E3.5. Error bars represent the mean ± SD (*n* = 3). **c** MANT dGDP and MANT GTP nucleotide exchange and release assays. SOS-mediated exchange of MANT-labelled dGDP or GTP on KRAS^G12V^ was studied over time upon addition of 10 μM concentrations of DARPins. Included controls were previously published RAS-binding DARPins K55 (non-inhibitor of nucleotide exchange) and K27 (inhibitor of nucleotide exchange). For release assays, DARPins were added after equilibration of MANT nucleotides. In these assays, the binding of MANT-labelled nucleotides to KRAS^G12V^ results in an increase in fluorescence, detected by measuring light emission at a wavelength of 450 nm. Error bars represent the mean ± SD (*n* = 8). **d** Superimposition of K19/KRAS^G12V^-GDP (PDB 6H47) and SOScat structures (PDB 1BKD)

While K13 and K19 bind to the allosteric lobe of KRAS and interfere with effector interactions, it suggested new mechanism(s) by which DARPins inhibit KRAS function. To this end, we tested these DARPins in biochemical assays, where K13 and K19 were compared with the known RAS nucleotide exchange inhibitor K27 and known RAS/CRAF RBD interaction inhibitor K55^8^. In vitro analyses show that DARPins K13 and K19 inhibit the biochemically coupled KRAS assay based on the inhibition of nucleotide exchange or KRAS–CRAF RBD interaction (Fig. 6a) as does K27 but, unlike K55, K13 and K19 do not prevent KRAS/CRAF RBD interaction in vitro (Fig. 6b) even though they do in the cell-based BRET assay (Fig. 4a). Furthermore, these DARPins lock down the GDP- and GTP-bound KRAS, thus preventing the nucleotide exchange by SOS (Fig. 6c). Nonetheless, the binding of the DARPins to KRAS is nucleotide independent (Fig. 3a; Supplementary Fig. 1a), and the binding occurs on a surface remote from the switch regions (Fig. 1). However, SOS not only contacts RAS near the switch 1 and 2 regions but also makes additional interactions with the residues 95–105 of the helix α3^29^. These contacts would cause a clash between K13 and SOScat for KRAS binding (Fig. 6d) and indicate why K13 and K19 inhibit the nucleotide exchange of KRAS.

**Fig. 7:**
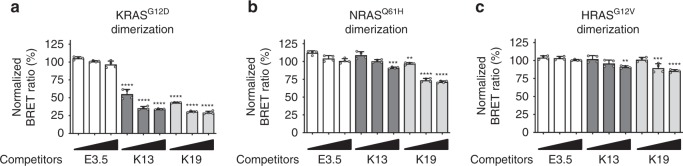
K13/K19 selectively prevent KRAS dimerisation. BRET competition assays with homodimerisation of (**a**) KRAS^G12D^; (**b**) of NRAS^Q61H^ and (**c**) of HRAS^G12V^ using DARPins K13 or K19 as competitors and DARPin E3.5 as non-binding negative control. Statistical analyses in **a**–**c** were performed using a one-way ANOVA followed by Dunnett’s post-hoc tests (**P < 0.01, ***P < 0.001, ****P < 0.0001). Each experiment was performed four times (**a**–**c**). Error bars are mean ± SD of biological repeats. **a**–**c** Source data are provided as a Source Data file

In addition, we investigated whether the DARPins could interfere with KRAS dimerisation, as it has been previously described with a different allosteric inhibitor, the monobody NS1^12^. Indeed, the α3–α4 and α4–α5 regions are defined as the potential dimer interfaces of KRAS^14–17^ and K13 and K19 interact with the α3–α4 site of KRAS (Supplementary Fig. 7a). Consequently, the binding of K13/K19 with KRAS is expected to perturb its dimerisation. We tested this hypothesis by BRET experiments involving the dimerisation of mutant K-, N- and H-RAS. K13 and K19 essentially inhibit KRAS^G12D^ dimerisation, while NRAS^Q61H^ and HRAS^G12V^ dimers formation is less affected by the DARPins (Fig. 7a–c).

It has been previously reported that the CAAX motif of RAS is mandatory for its dimerisation^18^. Therefore, we investigated RAS dimerisation using KRAS^G12V^ deleted of its last 22 amino acids (a KRAS^G12V^_166_ mutant missing the whole hypervariable region, HVR, including the CAAX motif) that should remove its ability to form RAS dimers. First, we showed that KRAS^G12V^_166_ was soluble and expressed in the cytoplasm of cells (Supplementary Fig. 10a) and was not able to form dimers in cells by BRET donor saturation assays (Supplementary Fig. 10b). We then performed a BRET competition assay with K13 and K19 against KRAS^G12V FL^ or KRAS^G12V^_166_ interaction with CRAF RBD showing that removal of the HVR from KRAS^G12V^ only reduced K13/K19 inhibition of KRAS^G12V^/CRAF RBD interaction (Supplementary Fig. 10c). These results show that RAS dimerisation in cells is not the only mechanism of inhibition of KRAS by the DARPins, as these retain the ability to inhibit KRAS^G12V^_166_/CRAF RBD interaction.

## Discussion

We reported here KRAS-selective inhibitors that bind to a previously untargeted site on the allosteric lobe of KRAS and prevent KRAS-mediated signalling. We have studied two DARPins, sharing a conserved amino acid sequence, that bind on the same interface of KRAS, the helix α3/loop 7/helix α4 region. The tryptophan residues 35 and 37 of K13 and K19 contact the histidine 95 of KRAS, which is a residue only found in KRAS thereby conveying their KRAS selectivity. These DARPins differ significantly from our previously isolated anti-RAS DARPins that included a pan-RAS-GTP binder (K55) that inhibits RAS/effector interactions and a pan-RAS-GDP binder (K27) that prevents RAS nucleotide exchange, both DARPins targeting the switch regions of RAS. We have now characterised K13/K19 DARPins that specifically contact KRAS (GTP and GDP bound) on an allosteric site remote from the switch regions and impede KRAS nucleotide exchange and dimerisation.

Even though K13/K19 bind to both mutant and WT KRAS, they only prevent RAS-dependent signalling in KRAS mutant cancer cells (HCT116) and do not affect RAS signalling in RAS^WT^ cancer cells (MCF-7) (Fig. 5). In a previous study, an antisense oligonucleotide (AZD4785) was described that selectively targets *KRAS* mRNA^30^, but both wild-type and mutant mRNAs. AZD4785 downregulates both WT and mutant KRAS protein, but only inhibits the downstream MAPK and PI3K signalling pathways of mutant KRAS expressing cells and not of KRAS^WT^ expressing cancer cells^30^. Therefore, our data also suggest that inhibition of both WT and mutant KRAS do not affect the RAS downstream signalling pathways of RAS^WT^ cancer cells. This effect could be explained by compensatory and/or redundancy mechanisms of the other RAS isoforms^31^.

Although the crystal structures of K13 and K19 bound to KRAS show substantial conformational change on both switch regions, the NMR HSQC and HDX-MS only show a small conformational change on the switch 2 region of KRAS upon the DARPins binding (Fig. 2 and Supplementary Figs. 3–6). In addition, K13/K19 do not prevent the interaction KRAS^G12V^/CRAF RBD in vitro (Fig. 6b), indicating that the conformational change of the switch 2 is not the main driver of KRAS function inhibition in cells. The mechanism by which K13 and K19 inhibit KRAS function is complex. KRAS dimerisation occurs through the α3–α4 and/or α4–α5 interfaces that are dimerisation interfaces of KRAS^14–17^. However, only the α4–α5 has been functionally tested in cells^11–13^. We now show that targeting α3–α4 interface also leads to KRAS function inhibition. The mechanism underlying this inhibition could rely on inhibition of RAF activation as described with a monobody^12^. The DARPin binding may also promote a certain degree of membrane occlusion of the effector-binding region of KRAS and impede the binding of effectors on the switch regions^32^. Nevertheless, because the GEF SOS contacts the α3 region of RAS^29^ (as well as switch 1 and 2), K13 and K19 presumably also impede KRAS nucleotide exchange by engaging the α3–α4 site. Therefore, it adds another level of complexity in the interpretation of the mechanisms responsible for KRAS inhibition. We conclude that K13/K19 can affect KRAS function by at least two different mechanisms, including nucleotide exchange and RAS dimerisation inhibition.

None of the mechanisms above would explain why DARPins K13 and K19 disrupt the binding of K27 to KRAS^WT^ in a BRET competition assay in cells (Supplementary Fig. 11a). This interaction should not rely on RAS dimerisation, since K27 interacts predominantly on the switch 1 of KRAS–GDP and no steric clash appears visible between K27 and K13 or K19 (Supplementary Fig. 11b, c). Therefore, the mechanism of inhibition of KRAS by K13 and K19 seems more complex than only the prevention of nucleotide exchange and KRAS dimerisation. While K27 only binds KRAS–GDP, K13/K19 also interact and lock down GTP-bound KRAS (Fig. 6c; Supplementary Fig. 1a). Hence, K13/K19 could inhibit the GTPase-activating proteins (GAP) binding on KRAS. The superimposition of the structures of two GAP proteins (p120 RAS GAP, PDB 1WQ1 and NF1, PDB 1NF1) with the structures of KRAS^G12V^-GDP/K13 (Supplementary Fig. 12a, b) and KRAS^G12V^-GDP/K19 (Supplementary Fig. 12c, d) shows that the amino terminal end of these DARPins would overlap with the bound GAP proteins, suggesting that K13/K19 may be GAP inhibitors. Even though a putative GAP inhibitory mechanism could explain the inhibition of KRAS^WT^/K27 interaction by K13/K19, it is not the mechanism that prevails in either mutant KRAS or KRAS^WT^ cancer cells. Indeed, RAS signalling is decreased in HCT116 cells (bearing both *mutant* and *WT KRAS* alleles), and is not increased in MCF-7 cells (homozygous for *KRAS*^*WT*^) when K13/K19 are expressed (Fig. 5). Nevertheless, HT1080 cells (bearing *NRAS*^*Q61K*^ and *KRAS*^*WT*^ alleles) expressing K19 present an increased level of pERK and pMEK signals (Fig. 5). This result could be assigned to the inhibition of the GAP binding on KRAS^WT^ by K19. However, this effect is not observed in T24 cells (bearing *HRAS*^*G12V*^ and *KRAS*^*WT*^ alleles), suggesting this increase of signalling might be cell line dependent. Therefore, the GAP inhibitory effect on RAS downstream signalling could be balanced by the dimerisation inhibition of KRAS^WT^ and/or by compensatory mechanisms involving for instance the two other RAS isoforms^31^ and explaining why RAS signalling is not always affected in KRAS^WT^ cancer cells, such as MCF-7.

The therapeutic use of these inhibitors with an intracellular protein such as KRAS is currently limited, but this work has identified an allosteric site on KRAS and the different inhibitory mechanisms involved in targeting this interface. Hence, the α3/loop 7/α4 region could be targeted for inhibition via alternative approaches. In future studies, the DARPins K13 and K19 could be used as surrogates to select small molecules targeting the α3/loop 7/α4 allosteric interface, especially around the KRAS selective residue H95. The surrogate method has been successfully used previously to isolate RAS-binding compounds using an anti-RAS intracellular single-domain antibody fragment^7^. Furthermore, an in silico study showed a potential small molecule-binding site on that interface^33^. This would be a promising way to isolate KRAS selective small inhibitors for the treatment of KRAS mutant cancers.

## Methods

### Isolation of DARPins K13 and K19

DARPins were isolated from a phage display library^34^ by panning selections on biotinylated KRAS^G12V^ (1–166) pre-bound to streptavidin magnetic beads, as previously described^8^. DARPin K19 was isolated from a first round phage display output that was further selected against KRAS^G12V^ (1–166) by ribosome display and error prone mutagenesis, using methods described by Groves et al.^35^ To convert a DARPin phage display library into a format compatible with ribosome display, primer DARPin-RD1 (5′- AGACCACAACGGTTTCCCTCTAGAAATAATTTTGTTTAACTTTAAGAAGGAGATATATCCATGGCCGATCTGGGAAA-3′) was used in place of primer SDCAT-DP47^35^.

For characterisation, DARPins were sub-cloned to the pET16b vector and expressed cytoplasmically in BL21 (DE3) *Escherichia coli* (New England Biolabs). A list of the primers used in this study is shown in Supplementary Table 3. Following lysis in BugBuster plus Benzonase (EMD Millipore), DARPins were purified to homogeneity using nickel-chelate chromatography, followed by size exclusion chromatography to provide a monomeric protein in PBS (pH 6.5).

### KRAS expression and purification

The human KRAS gene sequence (residues 1–166, Isoform 2B, P01116-2) containing the G12V substitution was cloned into a pET28b vector with an N-terminal His6 tag. KRAS^G12V^ was expressed in BL21 (DE3) *Escherichia coli* (New England Biolabs) and cell pellets resuspended in 50 mM HEPES (pH 7.4), 100 mM NaCl, 20 mM Imidazole, 2 mM TCEP and 2 mM MgCl_2_. Following sonication, the sample was centrifuged at 20,000 rpm and proteins purified from the supernatant by Ni-NTA chromatography on a 5 -mL HisTrap HP column (GE Life Sciences, cat # 17-5248-02). Following elution in 50 mM HEPES (pH 7.4), 500 mM NaCl, 400 mM Imidazole, 2 mM TCEP and 2 mM MgCl_2_, samples were further purified by size exclusion chromatography on a Superdex 75 column. Final buffer composition was 50 mM HEPES (pH 7.4), 100 mM NaCl, 2 mM MgSO_4_. KRAS^G12V^ was biotinylated via an Avi tag using BirA enzyme and then exchanged with either GDP or GTPγS, catalysed by SOS (Son of Sevenless). The GTPγS in the KRAS GTPγS had a tendency to be hydrolysed to GDP on storage, so the levels were determined by electrospray mass spectroscopy.

### X-ray structure

DARPins K13 and K19 were mixed with KRAS^G12V^-GDP in equimolar ratio with a final concentration of 16 mg.mL^−1^ and incubated on ice for 30 min prior to crystallisation screening at 277 and 293 K. Hits were observed in several conditions. Both K13 and K19 complexes gave bipyramidal crystals in 0.5–1.0 M lithium sulphate, 0.5–1.0 M ammonium sulphate, 100 mM tri-sodium citrate pH 5.5. A cryoprotectant solution of 2 M lithium sulphate could be used for crystals of both complexes.

The diffraction data were collected at 100 K temperature at the Diamond Light Source, UK on beamlines I04-1 and I03 at 0.920 and 0.976 Å wavelength, respectively. XDS, pointless and scala were used to process the data. Model building and subsequent refinement were carried out with Coot and Refmac. There are no Ramachandran outliers in either structures and over 98% are in the most favoured region. Coordinates and structure factors were deposited in the PDB with accession codes 6H46 and 6H47. Data collection and refinement statistics are summarised in Supplementary Table 1.

### NMR spectroscopy

Protein was expressed in BL21-Gold cells grown in M9 media supplemented with 5 g.L^−1^ Celtone N powder and containing 50 μg.mL^−1^ kanamycin and 15 μg.mL^−1^ tetracycline. Purification was performed as described above. Protein was dialysed overnight into NMR buffer (50 mM HEPES pH 7.4, 50 mM NaCl, 2 mM MgCl_2_, 2 mM TCEP, 0.1 mM EDTA, 0.02% NaN_3_)^36^. All NMR spectra were collected at 298 K on a Bruker 800 MHz, Avance III spectrometer, equipped with a 5 -mm TCI Cryoprobe with *z*-axis gradients using standard experiments and parameters from Bruker library. Uniformly 15-N-labelled KRAS^G12V^ was used at a concentration of 100 μM with concentrations of added non-labelled DARPins as indicated. NMR samples were prepared in HEPES-based buffer at pH 7.4 supplemented with 5% D_2_O. All spectra were processed in Topspin 3.1. Binding was monitored by 1H-15N 2D TROSY (transverse relaxation optimised spectroscopy) spectra of the protein acquired with (F2 × F1) 2048 × 160 complex pairs (in Echo-Antiecho mode), 12019 × 2757 Hz sweep width and 85.2 × 29.0 ms acquisition times. Assignments were mapped from deposited assignments of KRAS^WT^ BMRB ID: 18529 and HRAS^G12V^ BMRB ID: 25730.

### HDX mass spectrometry

Protein samples for HDX-MS were prepared in 20 mM HEPES–Na pH 7.4, 100 mM NaCl, 5 mM MgCl_2_, 1 mM TCEP at final protein concentrations of 10 μM GDP-loaded KRAS^G12V^ and 20 μM DARPin, and kept at 4 °C, unless otherwise stated. The deuterium exchange reactions were performed using a LEAP PAL RTC system (LEAP Technologies) and all conditions were performed in triplicate. Peptides were identified that covered 98.8% of the protein sequence; considering the rapid back-exchange of the two N-terminal residues, this reduces to 91.7%.

Deuterium exchange reactions were initiated by diluting the protein solutions 1:20 in 20 mM HEPES–Na pH 7.4, 100 mM NaCl, 5 mM MgCl_2_, 1 mM TCEP prepared in D_2_O (99.9% atom D, Sigma Aldrich), and incubating at 20 °C for 15 s, 60 s, 300 s, 900 s, 1800 s and 3600 s. Undeuterated controls were prepared by performing the same dilution but in buffer prepared in H_2_O. The labelling reactions were quenched by transferring 50 μL of the reaction mixture to 50 μL of pre-chilled quench solution (3.0 M urea, 1.6% (v/v) formic acid in water; 2 °C). Quenched samples were directly injected onto an Enzymate BEH immobilised pepsin column (2.1 × 300 mm, 3 mm; Waters) at 100 μL.min^−1^ at 20 °C for 3 min at 10,000 psi. Peptic peptides were trapped and desalted on an Acquity BEH C18 VanGuard pre-column (130 Å, 2.1 × 5 mm, 1.7 μm; Waters) kept at 0.1 °C. The trapped peptides were eluted using a 9 -min gradient of 5–35% acetonitrile in 0.1% (v/v) formic acid at 40 μL.min^−1^ on an Acquity UPLC BEH C18 column (130 Å, 1.7 μm, 100 mm × 1 mm; Waters) at 0.1 °C. Peptides were detected on a SYNAPT G2-Si HDMS mass spectrometer (Waters) acquiring over an m/z range of 50–2000 with an electrospray source and lock mass calibration (Leucine Enkephalin, 200 pg.μL^−1^; Waters). The mass spectrometer was operated at a source temperature of 80 °C and a spray voltage of 2.5 kV. Spectra were collected in positive ion resolution mode.

Peptide identification was performed in Protein Lynx Global Server (Waters) using MS^E^ data collected for the triplicate undeuterated control samples. The resultant peptide lists were imported into DynamX (Waters) where peptides were filtered: minimum intensity of 5000, minimum of 0.3 products per amino acid, a maximum MH^+^ error of 5 ppm, and found in all of the undeuterated data sets. The automatic peptide assignment in DynamX was performed using the standard parameters, but all peptides were manually checked for the charge-state assignment, overlapping peptides and retention time. The data were not corrected for back exchange, so are relative rather than absolute deuterium uptake values. The first two residues of each peptide were excluded from analyses due to rapid back exchange. Significant differences were determined by performing a Student’s *t* test using the pooled standard deviation or peptide standard deviation, whichever was the greater. The *p*-values were adjusted for false discovery using the Benjamini–Hochberg procedure. Differences with *p* < 0.05 were considered significant.

Peptide-level differences in deuterium uptake are calculated according to the Eq. (1):

1 $${\mathrm{pep}}_{\mathrm{i}}^{{\mathrm{state}}} = {\mathrm{uptake}}_{\mathrm{i}}^{{\mathrm{state}}} - {\mathrm{uptake}}_{\mathrm{i}}^{{\mathrm{apo}}}$$

pep_i_^state^: Difference in deuterium uptake for peptide i in state of interest

uptake_i_^state^: Deuterium uptake for peptide i in state of interest

uptake_i_^apo^: Deuterium uptake for peptide i apo state

The mean residue-level differences were calculated using the Eq. (2):

2 $${\mathrm{res}}_{\mathrm{j}} = \frac{1}{N}\mathop {\sum }\limits_{{\mathrm{i}} = 1}^{N} \frac{{{\mathrm{pep}}_{\mathrm{i}}}}{{{\mathrm{amide}}_{\mathrm{i}}}}$$

res_j_: mean difference for residue j

*N*: number of peptides containing residue j

pep_i_: deuterium uptake difference for peptide i that contains residue j

amide_i_: number of exchangeable residues within peptide i

### KRAS biochemical coupled assay and KRAS/CRAF RBD assay

The KRAS biochemical coupled assay was performed as previously described^8^. Briefly, biotinylated KRAS^G12V^ was pre-incubated with streptavidin–Europium chelate to form a complex, and in a separate reaction, CRAF RBD-GST (glutathione-S-transferase) was pre-incubated with anti-GST-XL665 to form a second complex. Test samples were incubated with the streptavidin:KRAS complex for 15 min before addition of GTPγS and SOS (to initiate nucleotide exchange) and the CRAF RBD:anti-GST complex. Final concentrations added were 2 nM biotinylated KRAS; 37.5 ng.mL^−1^ streptavidin–Europium; 12 nM CRAF RBD-GST; 2 mg.mL^−1^ anti-RAF GST XL665; 4 mM GTPγS and 2 mM SOS. In addition, the buffer for the CRAF RBD:anti-GST complex contained 0.1 mg.mL^−1^ BSA and 0.1 M potassium fluoride. After 1 h incubation, fluorescent resonance energy transfer (FRET) was measured on an Envision plate reader at emission wavelengths 620 and 665 nm. The ratio of these values was fitted using non-linear regression in the application Prism (GraphPad Software). An assay to measure only the inhibition of KRAS/CRAF RBD was like the coupled assay above, with the exception that test samples were added after the completion of the nucleotide exchange step.

### MANT nucleotide exchange and release assays

Exchange of GDP with fluorescent MANT-dGDP or fluorescent MANT-GTP on KRAS was studied in an assay previously described^8^. DARPins were tested for inhibition of nucleotide exchange by incubating at 10 μM with 500 nM KRAS^G12V^, 1 mM SOS and 500 nM MANT-dGDP or MANT-GTP (Biolog). Exchange was measured over time by the increase in fluorescence of MANT-dGDP or MANT-GTP upon binding to KRAS and detected using an Envision plate reader (PerkinElmer) using 340 nm/450 nm excitation/emission filters. The data were captured at times from zero to 60 min. For nucleotide release assays, DARPins were added after equilibration of MANT nucleotides.

### DARPin K_d_ measurements

ForteBio Octet Red384 was used to determine the binding kinetics of DARPins to KRAS. Concentrations of DARPin K19 from 15,000 nM to 6.2 nM or DARPin K13 from 3000 nM to 12.4 nM were added to sensors pre-loaded with biotinylated KRAS (1–166; wild-type; GDP or GTPγS), associated for 300 s and dissociated for 600 s by washing in buffer. For each sample, a reference well was included only containing buffer. The data were analysed using ForteBio data analysis. Reference wells were subtracted from sample wells, and 1:1 local fitting model was used to fit curves to sensorgrams and determine k_on_, k_off_ and K_d_ values. The experiments were carried out in kinetic buffer from ForteBio.

### Cell culture

HEK293T human embryonic kidney cells, HCT116 cells, HT1080 and MCF-7 cells were grown in the DMEM medium (Life Technologies), and T24 cells in the RPMI medium (Life Technologies). All cell lines were supplemented with 10% FBS (Sigma) and 1% penicillin/streptomycin (Life Technologies) and MCF-7 cells were also supplemented with 10 μg.mL^−1^ insulin (Sigma). Cells were grown at 37 °C with 5% CO_2_. The specific RAS mutations in the tumour cell lines were confirmed using genomic PCR, cloning the PCR products and DNA sequencing.

### Genotyping of RAS mutant cell lines

RNA was extracted from 5 × 10^6^ HCT116, HT1080, T24, and MCF-7 cells using the RNeasy Plus Mini Kit (Qiagen) according to the manufacturer’s instructions. cDNA was synthesised from 2 μg of RNA using SuperScript II Reverse Transcriptase (Invitrogen). Primers were designed to amplify KRAS, NRAS and HRAS DNA, and incorporate HindIII and BamHI restriction sites for sub-cloning:

KRAS_F: 5′-TAAGCAAAGCTTATGACTGAATATAAACTTGTGGTAG-3′

KRAS_R: 3′-GAAAATTAAAAAATGCATTATAATGTAAGGATCCTAAGCA-5′

NRAS_F: 5′-TAAGCAAAGCTTATGACTGAGTACAAACTGGTGGTGG-3′

NRAS_R: 3′-GGATTGCCATGTGTGGTGATGTAAGGATCCTAAGCA-5′

HRAS_F: 5′-TAAGCAAAGCTTATGACGGAATATAAGCTGGTGGTG-3′

HRAS_R: 3′-GCAAGTGTGTGCTCTCCTGAGGATCCTAAGCA-5′

DNA was amplified using Phusion High-Fidelity DNA Polymerase (New England Biolabs) and, following digestion with HindIII and BamHI, the DNA was cloned into pBlueScript II SK (+) (Stratagene). Plasmid DNA was prepared from individual DH5a transformants using a QIAprep Spin Miniprep Kit (QIAGEN). KRAS, NRAS and HRAS mutations were verified by Sanger sequencing (Source Bioscience) of at least six clones from each cell line. The RAS mutations in the cell lines were confirmed as KRAS^G13D^ (heterozygous) in HCT116, NRAS^Q61K^ (heterozygous) in HT1080, HRAS^G12V^ (homozygous) in T24 cells and RAS^WT^ in MCF-7 cells.

### Cells transfection

HEK293T cells were transfected with Lipofectamine 2000 (Thermo-Fisher, see the BRET2 section). HCT116, HT1080 and MCF-7 were seeded in six-well plates (670,000; 300,000; 250,000 cells per well, respectively). Cells were transfected 24 h later with 2.5 μg of pEF-DARPin-mCherry plasmid, 5 μL (HT1080 cells) or 8.75 μL (HCT116) or 6.25 μL (MCF-7) of Lipofectamine LTX and 2.5 μL of PLUS^TM^ Reagent (Thermo-Fisher) for another 24 h before western blot analysis. T24 cells were transfected with the Neon transfection system (Thermo-Fisher) following the manufacturer instructions. HCT116, HT1080 and T24 were transfected in duplicate for each condition (two wells per condition). Cells were pulled together before cell lysis (see the Western blot analysis section).

### Molecular cloning

The following full-length KRAS constructs have been produced elsewhere: KRAS^S17N^ and KRAS^WT^, all with carboxy terminal CAAX^23^. KRAS^H95Q^, KRAS^H95L^ and KRAS^E107D^ were produced by PCR site-directed mutagenesis using pEF-RLuc8-KRAS^WT^ as a template^23^. The truncated KRAS^G12V^_166_ (missing the 22 last carboxy-terminal amino acids corresponding to KRAS hypervariable region) was amplified by PCR using pEF-RLuc8-KRAS^G12V^-CAAX as a template and cloned between NotI/XbaI of the pEF-RLuc8-MCS and pEF-GFP^2^-MCS. Wild-type NRAS and HRAS were obtained by PCR site-directed mutagenesis using pEF-RLuc8-NRAS^Q61H^ and HRAS^G12V^, respectively, as templates^23^. All RAS cDNAs (KRAS mutants, KRAS^WT^, NRAS^Q61H^ and HRAS^G12V^-CAAX) were cloned between NotI/XbaI of the pEF-RLuc8-MCS, pEF-GFP^2^-MCS and pEF-3xFLAG-MCS plasmids.

CRAF RBD (1–149), PI3Kα RBD (161–315), PI3Kγ RBD (190–315), RALGDS RA (788–884) and full-length CRAF^S257L^ cloning into pEF-GFP^2^-MCS or pEF-MCS-GFP^2^ was described elsewhere^23^. DARPins were cloned between NcoI/XhoI of the pEF-MCS-GFP^2^ plasmid and between NcoI/XhoI of the pEF-MCS-mCherry plasmid. A list of the primers used in this study is shown in Supplementary Table 3.

### BRET2 titration curves and competition assays

For all BRET experiments (titration curves and competition assays), 650,000 HEK293T were seeded in each well of a six-well plates. After 24 h at 37 °C, cells were transfected with a total of 1.6 μg of DNA mix, containing the donor (RLuc8 plasmid) + acceptor (GFP^2^ plasmid) ± competitor (DARPin-mCherry plasmid), using Lipofectamine 2000 transfection reagent (Thermo-Fisher). Cells were detached 24 h later, washed with PBS and seeded in a white 96-well plate (clear bottom, PerkinElmer) in OptiMEM no-phenol red medium complemented with 4% FBS. Cells were incubated for an additional 20–24 h at 37 °C before the BRET assay reading. A step-by-step protocol is described elsewhere^37^.

### BRET2 measurements

BRET2 signal was determined immediately after addition of coelenterazine 400a substrate (10 μM final) to cells (Cayman Chemicals), using an Envision instrument (2103 Multilabel Reader, PerkinElmer) with the BRET2 Dual Emission optical module (515–30 and 410–80 nm; PerkinElmer). The total GFP^2^ fluorescence was detected with excitation and emission peaks set at 405 and 515 nm, respectively. The total mCherry fluorescence was detected with excitation and emission peaks set at 530 and 615 nm, respectively. The total RLuc8 luminescence was measured with the luminescence 400–700 nm-wavelength filter.

The BRET signal or BRET ratio corresponds to the light emitted by the GFP^2^ acceptor constructs (515–30 nm) upon addition of coelenterazine 400a divided by the light emitted by the RLuc8 donor constructs (410–80 nm). The background signal is subtracted from that BRET ratio using the donor-only negative control, where only the RLuc8 plasmid is transfected into the cells. The normalised BRET ratio is the BRET ratio normalised to a negative control (DARPin control) during a competition assay. The total GFP^2^, mCherry and RLuc8 signals were used to control the protein expression level from each plasmid.

### Immunoprecipitation assay

HEK293T cells were transfected 48 h in duplicate (two wells per condition) with pEF-3xFLAG-RAS and pEF-DARPins-GFP^2^ plasmids. Cells were washed once with PBS and lysed in the immunoprecipitation buffer (150 mM NaCl, 50 mM Tris-HCl pH 7.4, 10 mM MgCl_2_, 10% glycerol and 0.5% Triton) supplemented with protease inhibitors (Sigma) and phosphatase inhibitors (Thermo-Fisher) for 20 min. Lysates were centrifuged for 15 min, and the supernatant incubated with protein G magnetic beads (Life Technologies) and anti-FLAG antibody (Sigma). The complexes were incubated 4 h at 4 °C on a wheel. Beads were washed five times with the IP buffer, the bound proteins were eluted with 1× loading buffer and resolved on 12.5% SDS-PAGE.

### Western blot analysis

Cells were washed once with PBS and lysed in SDS-Tris buffer (1% SDS, 10 mM Tris-HCl pH 7.4) supplemented with protease inhibitors (Sigma) and phosphatase inhibitors (Thermo-Fisher). Cell lysates were sonicated with a Branson Sonifier, and the protein concentrations determined by using the Pierce BCA protein assay kit (Thermo-Fisher). Equal amounts of protein were resolved on 10 or 15% SDS-PAGE and subsequently transferred onto a PVDF membrane (GE). The membrane was blocked either with 10% non-fat milk (Sigma) or 10% BSA (Sigma) in TBS-0.1% Tween20 and incubated overnight with primary antibody at 4 °C. After washing, the membrane was incubated with HRP-conjugated secondary antibody for 1 h at room temperature (RT, 25 °C). The membrane was washed with TBS-0.1% Tween and developed using Pierce ECL Western Blotting Substrate (Thermo-Fisher) and CL-XPosure films (Thermo-Fisher). Primary antibodies include anti-phospho-p44/22 MAPK (ERK1/2) (1/3000, CST, Cat#9101 S), anti-p44/42 MAPK (total ERK1/2) (1/1000, CST, Cat#9102 S), anti-phospho-MEK1/2 (1/1500, CST, Cat#9154 S), anti-MEK1/2 (1/500, CST, Cat#4694 S), anti-phospho-AKT S473 (1/1000, CST, Cat#4058 S), anti-phospho-AKT T308 (1/1000, CST, Cat#4056 S), anti-AKT (1/1000, CST, Cat#9272 S), anti-pan-RAS (1/200, Millipore, Cat#OP40), anti-GFP (1/500, Santa Cruz Biotechnologies, Cat#sc-9996), anti-DsRed (1/200, Santa Cruz Biotechnologies, Cat#sc-33353), anti-FLAG (1/2000, Sigma, Cat#F3165), anti-β-actin (1/2500, Sigma, Cat#A1978) and anti-α-tubulin (1/2000, Abcam, Cat#ab4074). Secondary antibodies include anti-mouse IgG HRP-linked (CST), anti-rabbit IgG HRP-linked (CST) and anti-goat IgG HRP-linked (Santa Cruz Biotechnologies).

### Confocal microscopy

HEK293T cells were seeded on coverslips and transfected 24 h with pEF-GFP^2^-KRAS^G12V^_166_ construct. Coverslips were washed in PBS, fixed 10 min in 4% paraformaldehyde and washed twice in PBS. Then the coverslips were mounted with DAPI Fluoromount-G overnight. Slides were analysed using a Zeiss 880 Inverted Confocal Microscope with a ×63 objective. Confocal images were analysed with ImageJ software.

### Quantification and statistical analysis

The quantifications were performed using ImageJ or Prism 7.0c (GraphPad Software), BRET titration curves and statistical analysis were performed using Prism 7.0c (GraphPad Software). The data are typically presented as mean ± SD or SEM as specified in the figure legends. Statistical analyses were performed with a one-way ANOVA followed by Dunnett’s post hoc tests, unless otherwise indicated in the figure legends. *P < 0.05, **P < 0.01, ***P < 0.001, ****P < 0.0001.

### Reporting summary

Further information on research design is available in the Nature Research Reporting Summary linked to this article.

## Supplementary information

Supplementary Information

Reporting Summary

Source Data

## Data Availability

Structure files and coordinates have been deposited to PDB under these accession numbers: 6H46 and 6H47. All relevant data are within the paper and its Supplementary Information file and the Source data file. The source data underlying Figs. 3c, 4a–d, 5a, b, 7a–c and Supplementary Figs. 7b, 9a, b, 10c and 11a are provided as a Source Data file. Additional data supporting the conclusions are available from the corresponding author on reasonable request.
