# Supplementary material for: KRAS-specific inhibition using a DARPin binding to a site in the allosteric lobe
Source: Nat Commun. 2019 Jun 13;10:2607. doi: 10.1038/s41467-019-10419-2 (PMC6565726; doi:10.1038/s41467-019-10419-2)
Supplement: Supplementary file 1 — Supplementary Information [file 41467_2019_10419_MOESM1_ESM.pdf]

Supplementary Information

KRAS-specific inhibition using a DARPin binding to a site in the allosteric lobe

Bery, N et al.

a

DARPin	Target	k_{on} $M^{-1}.s^{-1}$	k_{off} s^{-1}	K_d nM
K13	KRAS-GDP	$1.7 \times 10^5 \pm 0.3$	$6.1 \times 10^{-3} \pm 0.07$	39.9 ± 1.7
K13	KRAS-GTPγS	$4.3 \times 10^5 \pm 0.4$	$9.2 \times 10^{-3} \pm 0.3$	30.6 ± 3.2
K19	KRAS-GDP	$2.5 \times 10^5 \pm 0.3$	$1.4 \times 10^{-4} \pm 0.08$	10.8 ± 1.4
K19	KRAS-GTPγS	$2.5 \times 10^5 \pm 0.6$	$2.5 \times 10^{-4} \pm 0.04$	9.9 ± 1.9

b **K13 DNA and protein sequences**

```
         10        20        30        40        50        60        70        80        90       100
ATGGATCTGGGAAAAAAACTGCTGGAAGCCGCGCGTGCCGGGCAGGACGATGAGGTCCGTATTCTTATGGCGAACGGTGCAGATGTTAACGCGAGCGATC
TACCTAGACCCTTTTTTTGACGACCTTCGGCGCGCACGGCCCGTCCTGCTACTCCAGGCATAAGAATACCGCTTGCCACGTCTACAATTGCGCTCGCTAG
 M  D  L  G  K  K  L  L  E  A  A  R  A  G  Q  D  D  E  V  R  I  L  M  A  N  G  A  D  V  N  A  S  D>

        110       120       130       140       150       160       170       180       190       200
GCTGGGGTTGGACGCCGCTGCATCTGGCAGCGTGGTGGGGTCACCTCGAAATTGTGGAAGTGCTGTTGAAGCACGGTGCAGATGTTAACGCGGCAGATCT
CGACCCCAACCTGCGGCGACGTAGACCGTCGCACCACCCCAGTGGAGCTTTAACACCTTCACGACAACTTCGTGCCACGTCTACAATTGCGCCGTCTAGA
R  W  G  W  T  P  L  H  L  A  A  W  W  G  H  L  E  I  V  E  V  L  L  K  H  G  A  D  V  N  A  A  D  L>
   *     *                                                              *              *
        210       220       230       240       250       260       270       280       290       300
GCACGGTCAAACGCCGCTGCATCTGGCAGCGATGGTCGGTCACCTCGAAATTGTGGAAGTGCTGTTGAAGTACGGTGCAGATGTTAACGCGAAAGATACG
CGTGCCAGTTTGCGGCGACGTAGACCGTCGCTACCAGCCAGTGGAGCTTTAACACCTTCACGACAACTTCATGCCACGTCTACAATTGCGCTTTCTATGC
  H  G  Q  T  P  L  H  L  A  A  M  V  G  H  L  E  I  V  E  V  L  L  K  Y  G  A  D  V  N  A  K  D  T>
           *
        310       320       330       340       350       360       370       380       390       400
ATGGGTGCAACGCCGCTGCATCTGGCAGCGCAAAGCGGTCACCTCGAAATTGTGGAAGTGCTGTTGAAGAACGGTGCAGATGTGAATGCTCAGGATAAGT
TACCCACGTTGCGGCGACGTAGACCGTCGCGTTTCGCCAGTGGAGCTTTAACACCTTCACGACAACTTCTTGCCACGTCTACACTTACGAGTCCTATTCA
 M  G  A  T  P  L  H  L  A  A  Q  S  G  H  L  E  I  V  E  V  L  L  K  N  G  A  D  V  N  A  Q  D  K>
                               *                          *                       *
        410       420       430       440       450       460       470
TTGGCAAAACCGCGTTTGATATCTCCATTGATAATGGCAACGAAGATTTAGCGGAAATCCTGCAGAAACTG
AACCGTTTTGGCGCAAACTATAGAGGTAACTATTACCGTTGCTTCTAAATCGCCTTTAGGACGTCTTTGAC
F  G  K  T  A  F  D  I  S  I  D  N  G  N  E  D  L  A  E  I  L  Q  K  L>
            *              *
```

c **K19 DNA and protein sequences**

```
         10        20        30        40        50        60        70        80        90       100
ATGGATCTGGGAAAAAAACTGCTGGAAGCCGCGCGTGCCGGGCAGGACGATGAGGTCCGTATTCTTATGGCGAATGGTGCAGATGTTAACGCGAGCGATC
TACCTAGACCCTTTTTTTGACGACCTTCGGCGCGCACGGCCCGTCCTGCTACTCCAGGCATAAGAATACCGCTTACCACGTCTACAATTGCGCTCGCTAG
 M  D  L  G  K  K  L  L  E  A  A  R  A  G  Q  D  D  E  V  R  I  L  M  A  N  G  A  D  V  N  A  S  D>

        110       120       130       140       150       160       170       180       190       200
GTTGGGGTTGGACGCCGCTGCACCTGGCAGCGTGGTGGGGTCACCTCGAAATTGTGGAAGTGCTGTTGAAGCGCGGTGCAGATGTTAGCGCGGCAGATCT
CAACCCCAACCTGCGGCGACGTGGACCGTCGCACCACCCCAGTGGAGCTTTAACACCTTCACGACAACTTCGCGCCACGTCTACAATCGCGCCGTCTAGA
R  W  G  W  T  P  L  H  L  A  A  W  W  G  H  L  E  I  V  E  V  L  L  K  R  G  A  D  V  S  A  A  D  L>
   *     *                                                              *              *
        210       220       230       240       250       260       270       280       290       300
GCACGGTCAATCGCCGCTGCATCTGGCAGCGATGGTCGGCCACCTCGAAATTGTGGAAGTGCTGTTGAAGTACGGTGCAGATGTTAACGCGAAAGATACG
CGTGCCAGTTAGCGGCGACGTAGACCGTCGCTACCAGCCGGTGGAGCTTTAACACCTTCACGACAACTTCATGCCACGTCTACAATTGCGCTTTCTATGC
  H  G  Q  S  P  L  H  L  A  A  M  V  G  H  L  E  I  V  E  V  L  L  K  Y  G  A  D  V  N  A  K  D  T>
           *
        310       320       330       340       350       360       370       380       390       400
ATGGGTGCAACGCCGCTGCACCTGGCAGCGCGAAGCGGTCACCTCGAAATTGTGGAAGAGCTGTTGAAGAACGGTGCAGATATGAATGCTCAGGATAAGT
TACCCACGTTGCGGCGACGTGGACCGTCGCGCTTCGCCAGTGGAGCTTTAACACCTTCTCGACAACTTCTTGCCACGTCTATACTTACGAGTCCTATTCA
 M  G  A  T  P  L  H  L  A  A  R  S  G  H  L  E  I  V  E  E  L  L  K  N  G  A  D  M  N  A  Q  D  K>
                               *                          *                       *
        410       420       430       440       450       460       470
TTGGCAAAACCACGTTTGATATCTCCACTGATAATGGCAACGAAGATTTAGCGGAAATCCTGCAGAAACTG
AACCGTTTTGGTGCAAACTATAGAGGTGACTATTACCGTTGCTTCTAAATCGCCTTTAGGACGTCTTTGAC
F  G  K  T  T  F  D  I  S  T  D  N  G  N  E  D  L  A  E  I  L  Q  K  L>
            *              *
```

Supplementary Figure 1: determination of K13/K19 affinity and the corresponding nucleotide and amino acid sequences of K13 and K19 DARPins. (a) K_d of K13/K19 on KRAS-GDP and KRAS-GTPγS. (b, c) Nucleotide and protein sequences of DARPins K13 (b) and K19 (c). The black stars show the two tryptophan residues mutated in the K13/K19 mutants (hereafter named Mut). The light purple stars show the amino acid difference between K13 and K19 sequences. Pink amino acids: repeat 1, light blue amino acids: repeat 2 and orange amino acid: repeat 3.

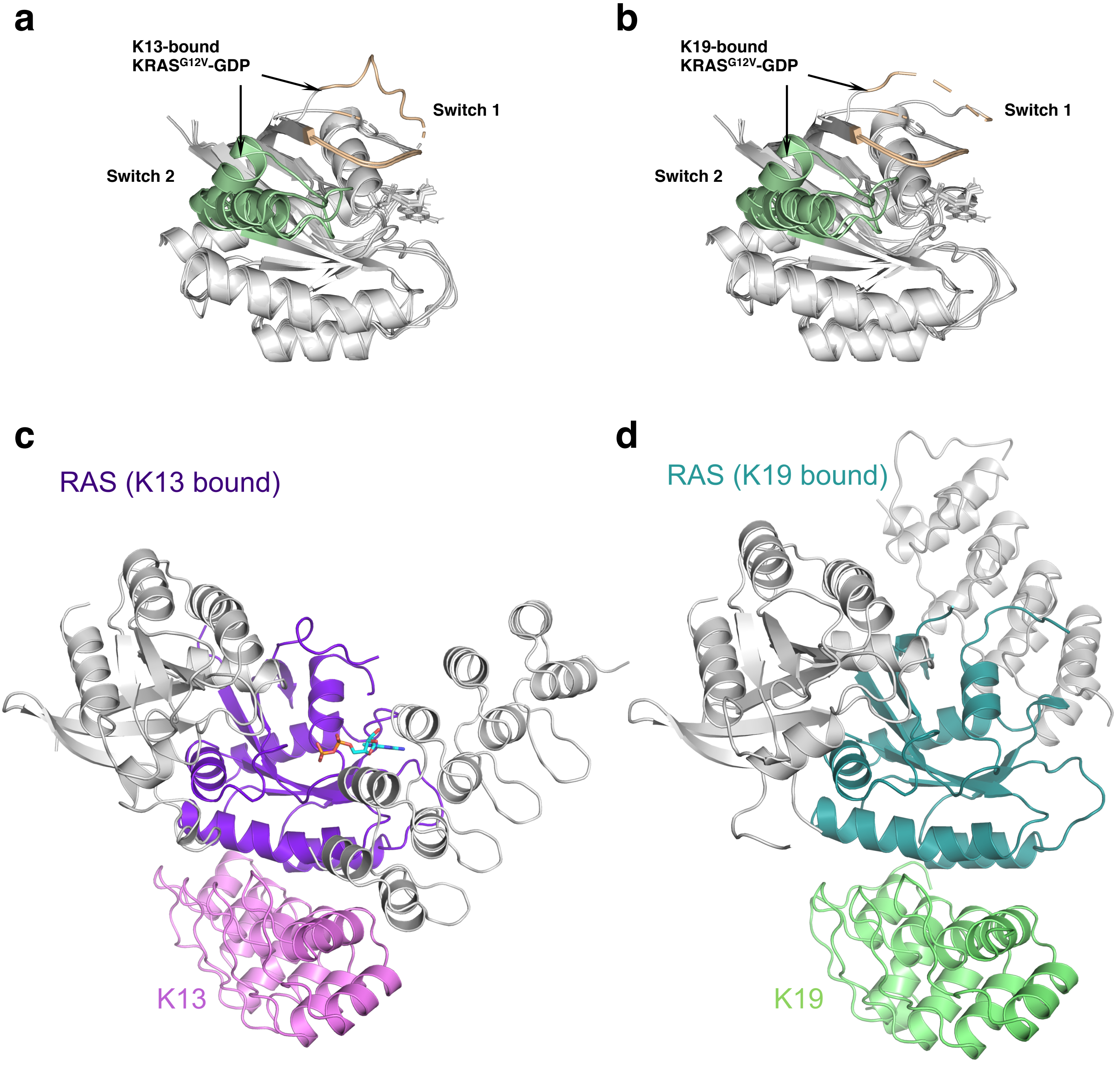

Supplementary Figure 2: Crystal packing effects on the change of conformation of the switch regions of KRAS upon DARPins binding. (a, b) Superimposition of KRAS^{G12V}-GDP structures (PDB 4TQ9 and 5UQW) with KRAS^{G12V}-GDP in complex with DARPins K13 (**a**, PDB 6H46) and K19 (**b**, PDB 6H47) using Pymol. KRAS^{G12V}-GDP is in grey with switch 1 and switch 2 regions highlighted in brown and green respectively. **(c, d)** Crystal packing environments around the K13 (c) and K19 (d) bound KRAS switch 1 loop show significant differences: the K19 forms is more restricted by symmetry equivalent molecules packing in the proximity of this loop. Symmetry equivalent molecules that are in direct contact with the KRAS molecules are drawn in grey.

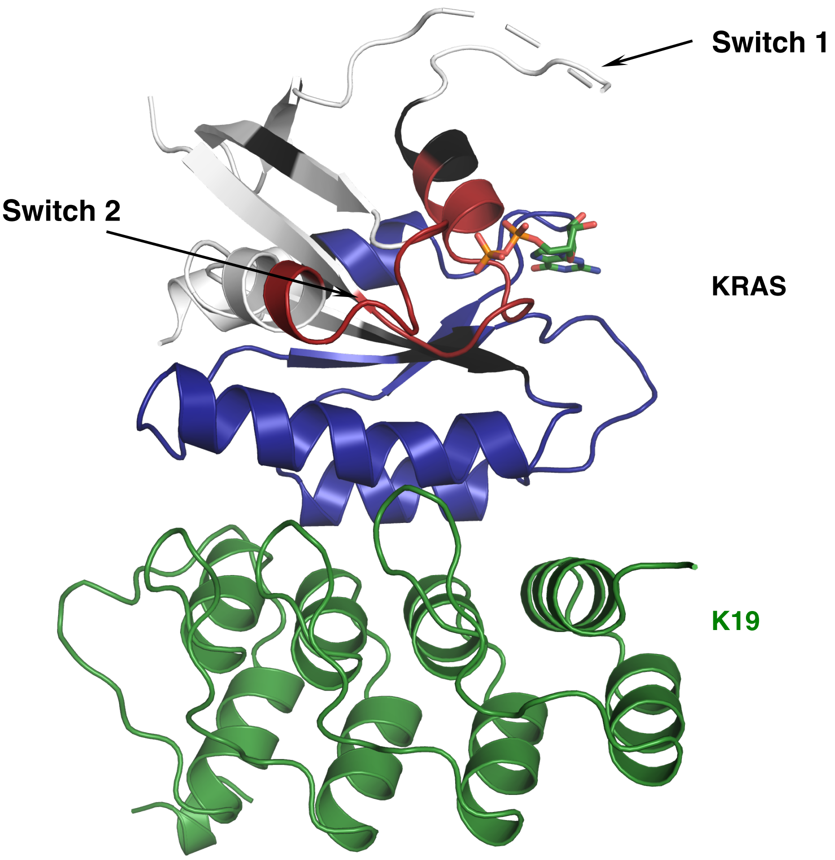

Supplementary Figure 3: KRAS HDX-MS heat map. HDX protection differences mapped onto the KRAS^{G12V}-GDP crystal structure (PDB 4TQ9) upon K19 binding. Regions of KRAS showing increased protection are coloured blue, regions of KRAS showing increased de-protection are shown in red. Residues that do not have coverage in the HDX analysis are shown in black. Increased protection indicates regions that are less solvent accessible upon DARPin binding and increased de-protection indicates regions that are more solvent accessible upon DARPin binding. The switch regions are indicated on KRAS structure. DARPin K19 is coloured in green.

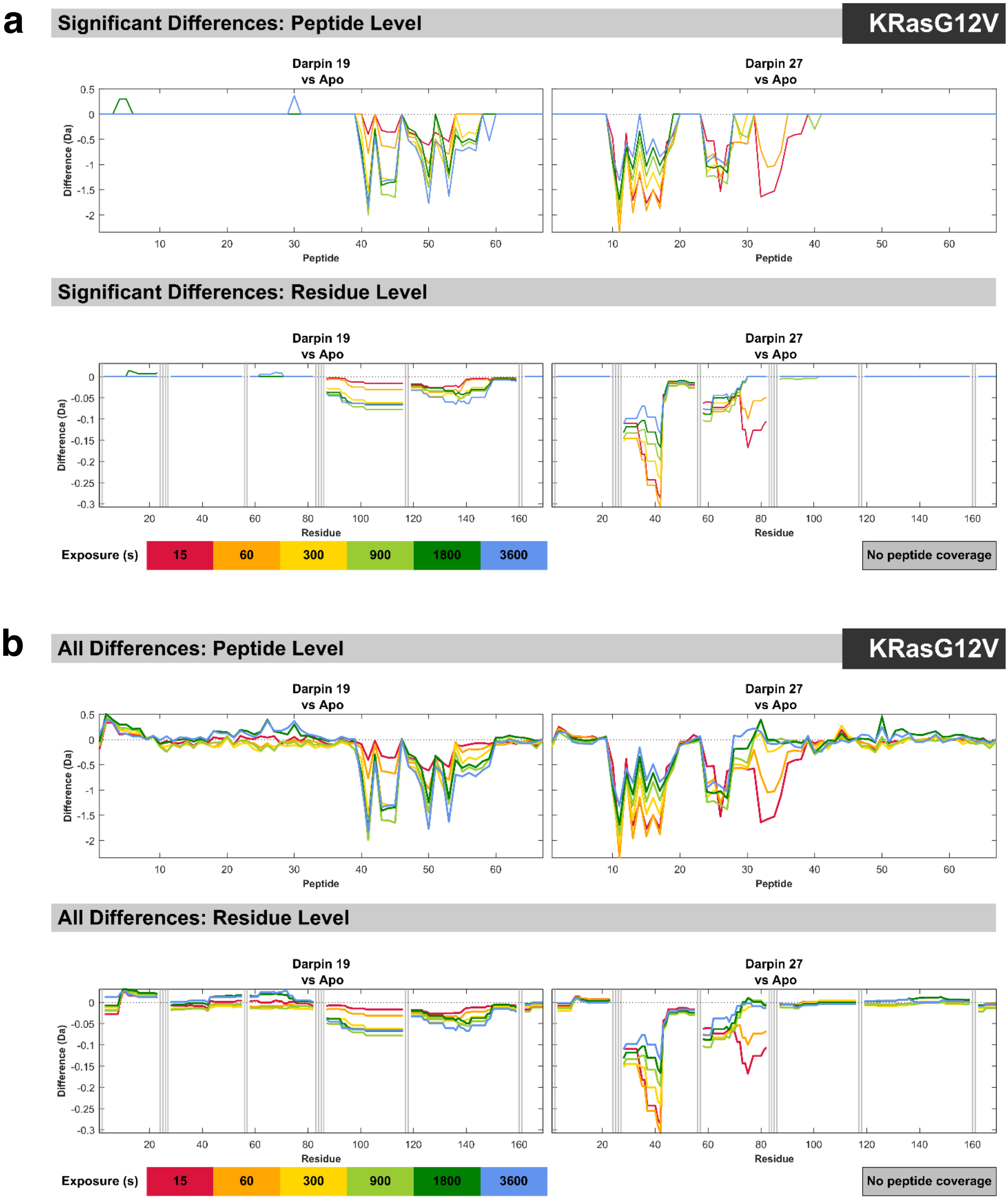

Supplementary Figure 4: HDX-MS average difference in deuterium uptake over all time points. (a) HDX data after filtering for statistical significance (adjusted p-value < 0.05). Upper panel: The peptide-level difference in deuterium uptake between DARPin-bound and apo-KRAS^{G12V} (calculated by subtracting the Apo-KRAS protein data from the DARPin-bound KRAS data for each peptide). Lower panel: The difference in deuterium uptake for each residue is shown, calculated by taking the weighted average of uptake differences over relevant overlapping peptides. **(b)** HDX data for all peptides (i.e. no statistical testing). Upper panel: The peptide-level difference in deuterium uptake between DARPin-bound and apo-KRAS^{G12V} (calculated by subtracting the Apo-KRAS protein data from the DARPin-bound KRAS data for each peptide). Lower panel: The difference in deuterium uptake for each residue, calculated by taking the weighted average of uptake differences of relevant overlapping peptides.

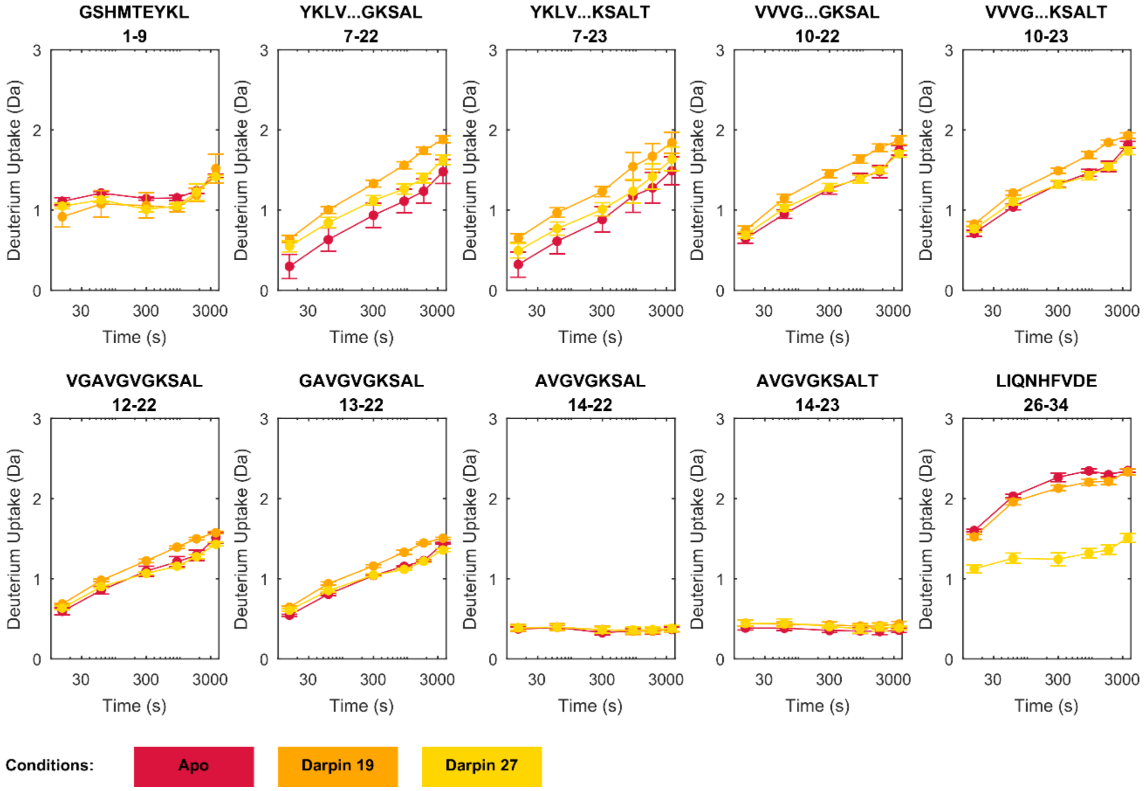
GSHMTEYKL
1-9
YKLV...GKSAL
7-22
YKLV...KSALT
7-23
VVVG...GKSAL
10-22
VVVG...KSALT
10-23
VGAVGVGKSAL
12-22
GAVGVGKSAL
13-22
AVGVGKSAL
14-22
AVGVGKSALT
14-23
LIQNHFVDE
26-34
Deuterium Uptake (Da)
Time (s)
Conditions:
Apo
Darpin 19
Darpin 27

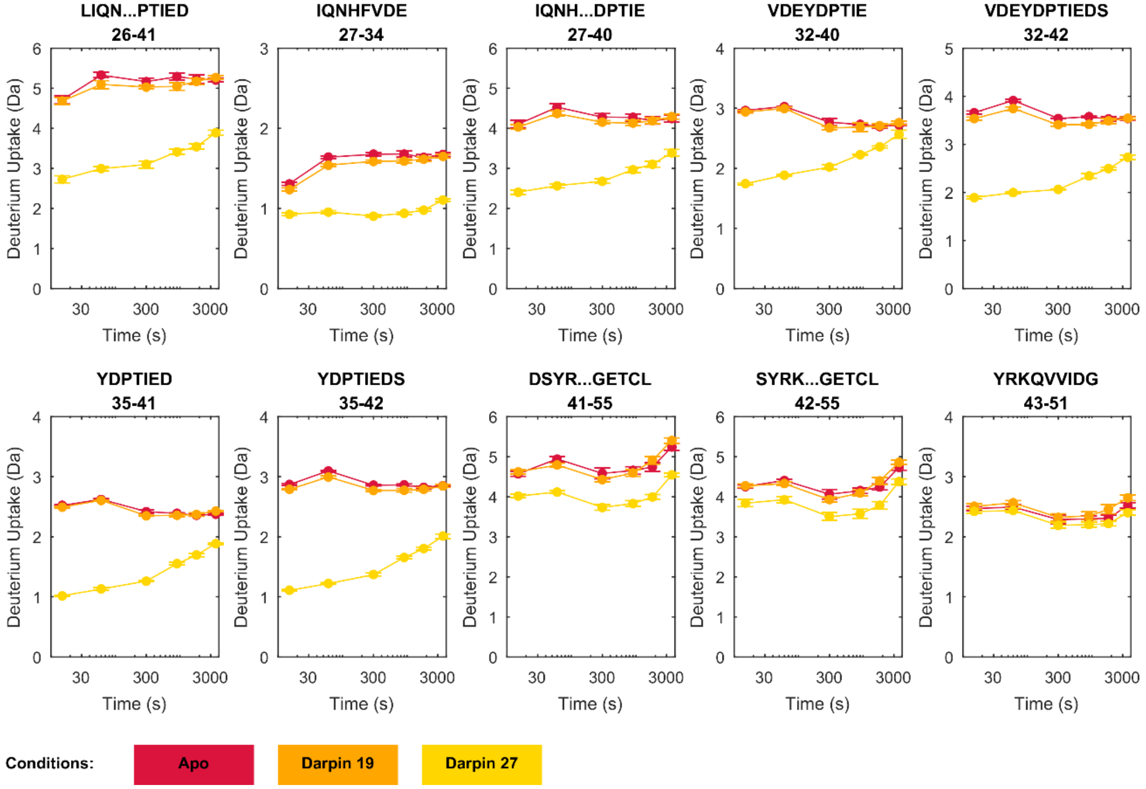
LIQN...PTIED
26-41
IQNHFVDE
27-34
IQNH...DPTIE
27-40
VDEYDPTIE
32-40
VDEYDPTIEDS
32-42
YDPTIED
35-41
YDPTIEDS
35-42
DSYR...GETCL
41-55
SYRK...GETCL
42-55
YRKQVVIDG
43-51
Deuterium Uptake (Da)
Time (s)
Conditions:
Apo
Darpin 19
Darpin 27

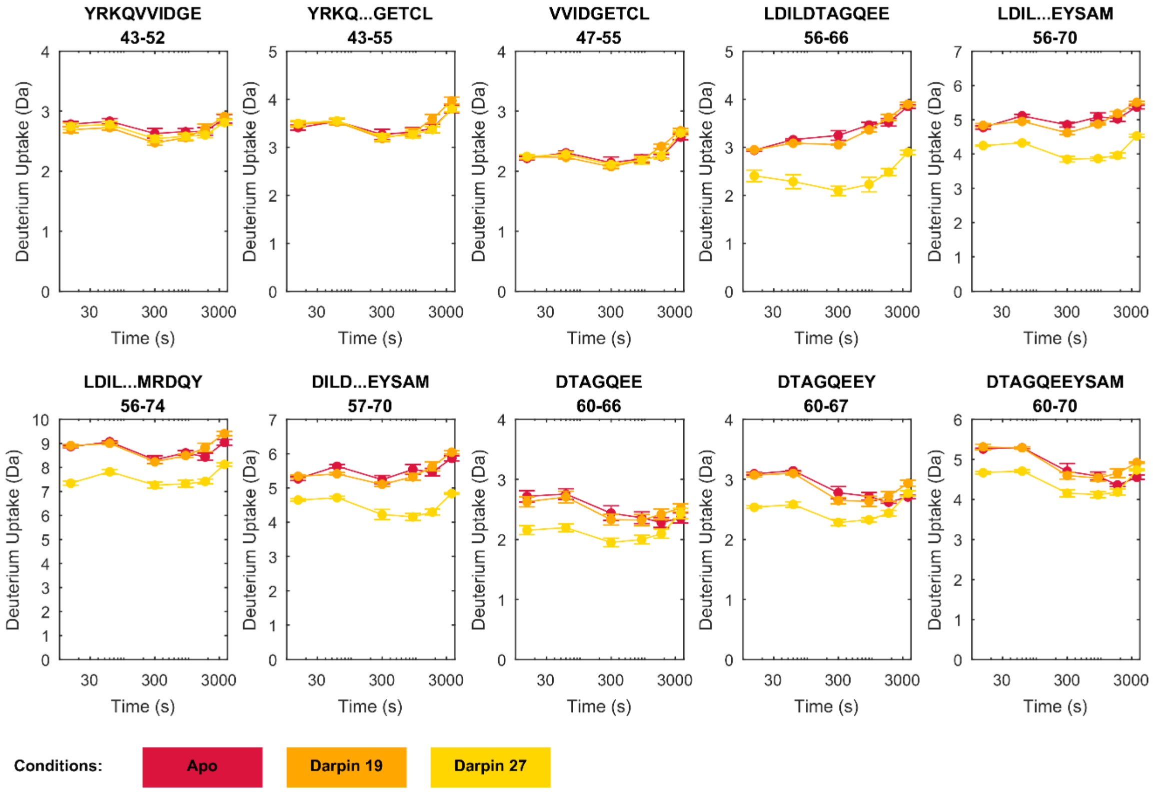
YRKQVVIDGE
43-52
YRKQ...GETCL
43-55
VVIDGETCL
47-55
LDILDTAGQEE
56-66
LDIL...EYSAM
56-70
LDIL...MRDQY
56-74
DILD...EYSAM
57-70
DTAGQEE
60-66
DTAGQEEY
60-67
DTAGQEEYSAM
60-70
Deuterium Uptake (Da)
Time (s)
Conditions:
Apo
Darpin 19
Darpin 27

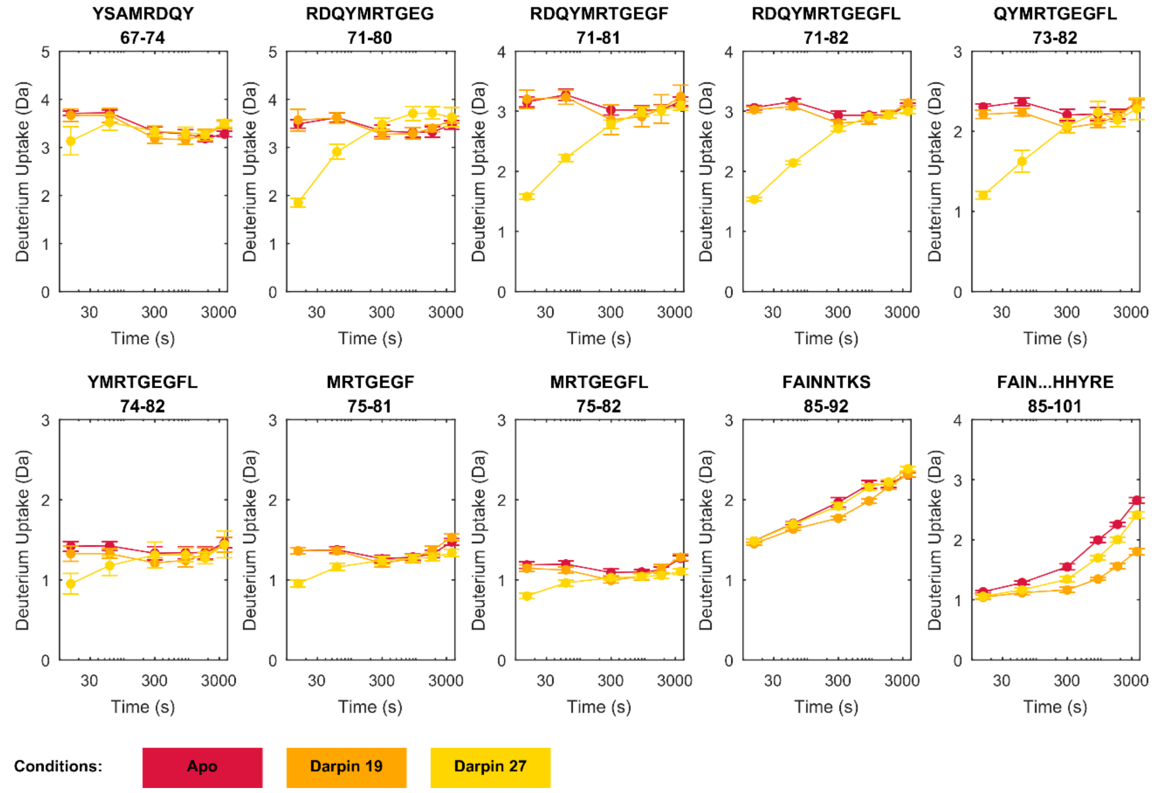
YSAMRDQY
67-74
RDQYMRTGEG
71-80
RDQYMRTGEGF
71-81
RDQYMRTGEGFL
71-82
QYMRTGEGFL
73-82
YMRTGEGFL
74-82
MRTGEGF
75-81
MRTGEGFL
75-82
FAINNTKS
85-92
FAIN...HHYRE
85-101
Deuterium Uptake (Da)
Time (s)
Conditions:
Apo
Darpin 19
Darpin 27

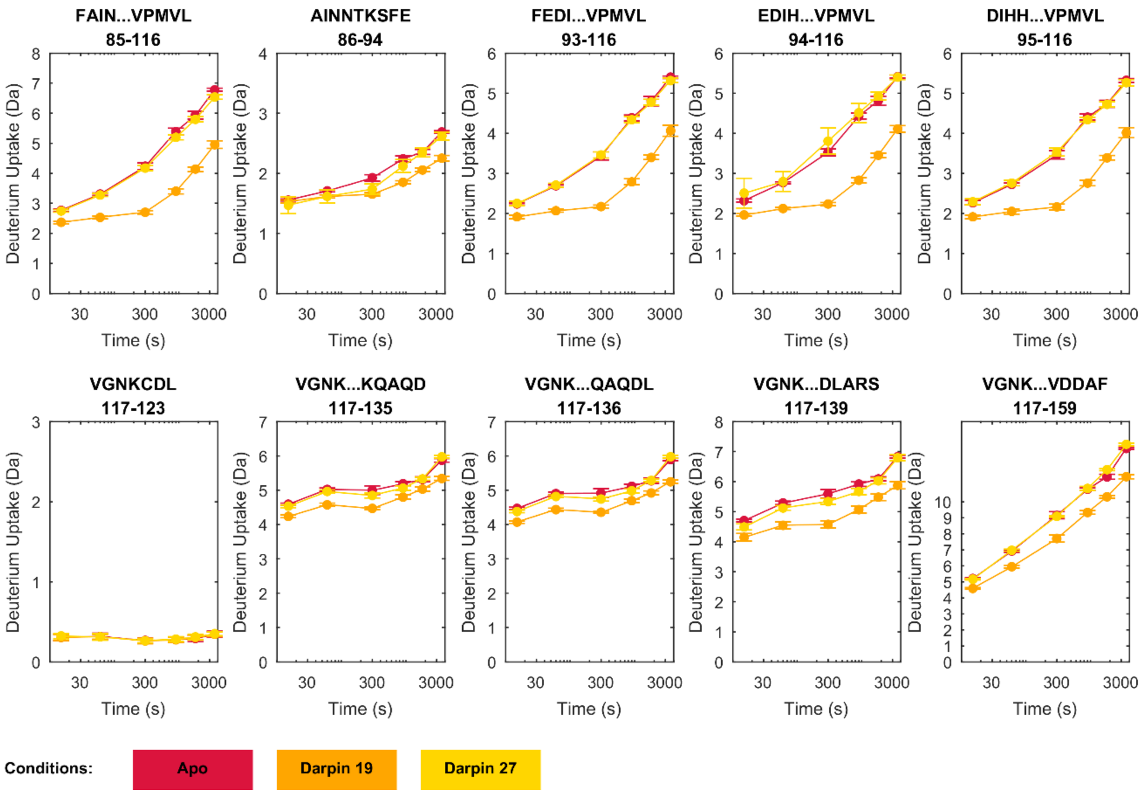
FAIN...VPMVL
85-116
AINNTKSFE
86-94
FEDI...VPMVL
93-116
EDIH...VPMVL
94-116
DIHH...VPMVL
95-116
VGNKCDL
117-123
VGNK...KQAQD
117-135
VGNK...QAQDL
117-136
VGNK...DLARS
117-139
VGNK...VDDAF
117-159
Deuterium Uptake (Da)
Time (s)
Conditions:
Apo
Darpin 19
Darpin 27

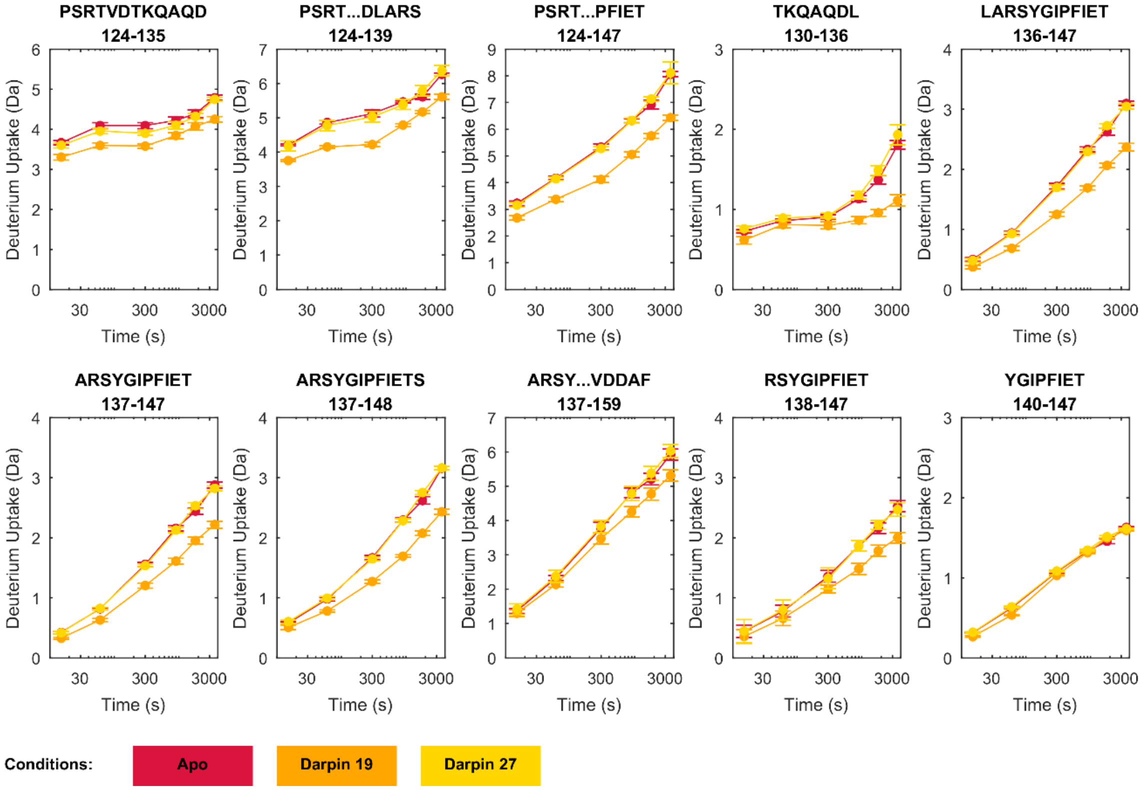
PSRTVDTKQAQD
124-135
PSRT...DLARS
124-139
PSRT...PFIET
124-147
TKQAQDL
130-136
LARSYGIPFIET
136-147
ARSYGIPFIET
137-147
ARSYGIPFIETS
137-148
ARSY...VDDAF
137-159
RSYGIPFIET
138-147
YGIPFIET
140-147
Deuterium Uptake (Da)
Time (s)
Conditions:
Apo
Darpin 19
Darpin 27

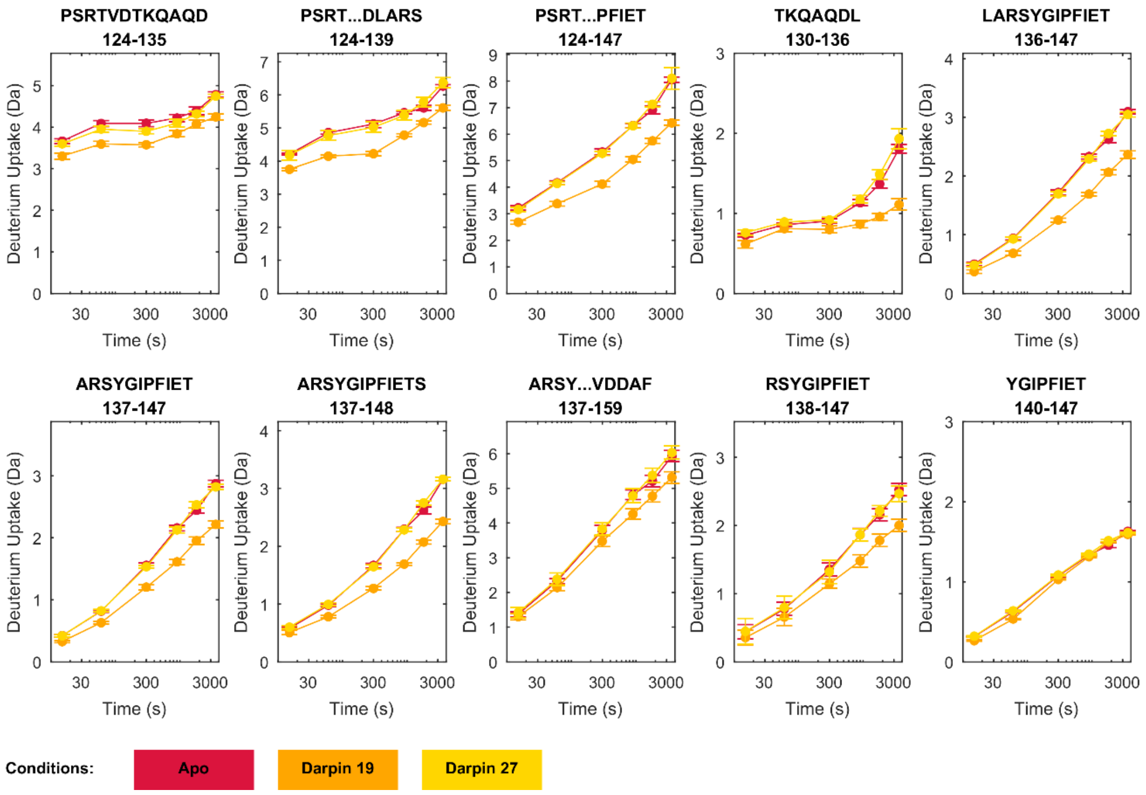

Supplementary Figure 5: HDX-MS relative deuterium uptake plots. Level of deuterium incorporation for each peptide, in each condition as a function of time. The error bars represent one standard deviation around the mean of three independent experiments (biological repeats).

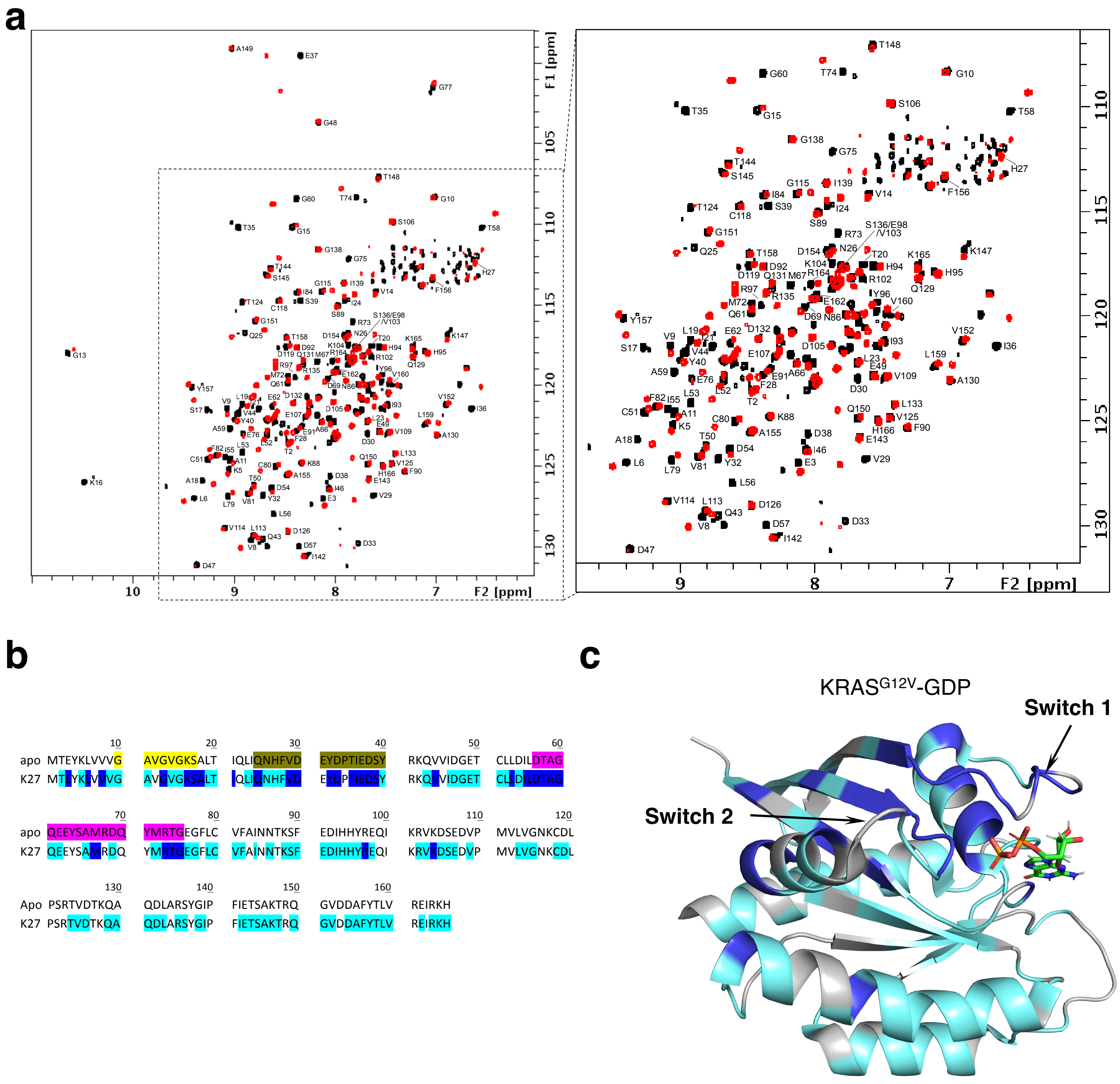

Supplementary Figure 6: NMR chemical shift changes in KRAS upon K27 binding. (a) Comparison between ^{1}H, ^{15}N TROSY spectra for apo KRAS^{G12V} at 100 μM (black) overlaid with K27 bound KRAS^{G12V} at 91 μM (red). DARPin concentrations were in slight excess. An expanded region is shown on the right. **(b)** Amino acids undergoing chemical shifts upon K27 binding on KRAS^{G12V}-GDP are shown in blue, residues not experiencing shifts are shown in turquoise in the KRAS^{G12V} sequence (unassigned residues are not highlighted). **(c)** Ribbon representation of (b) with amino acids undergoing chemical shifts in blue and residues not experiencing shifts in turquoise in the KRAS^{G12V}-GDP structure (PDB 4TQ9) after DARPin K27 binding (unassigned residues are in grey).

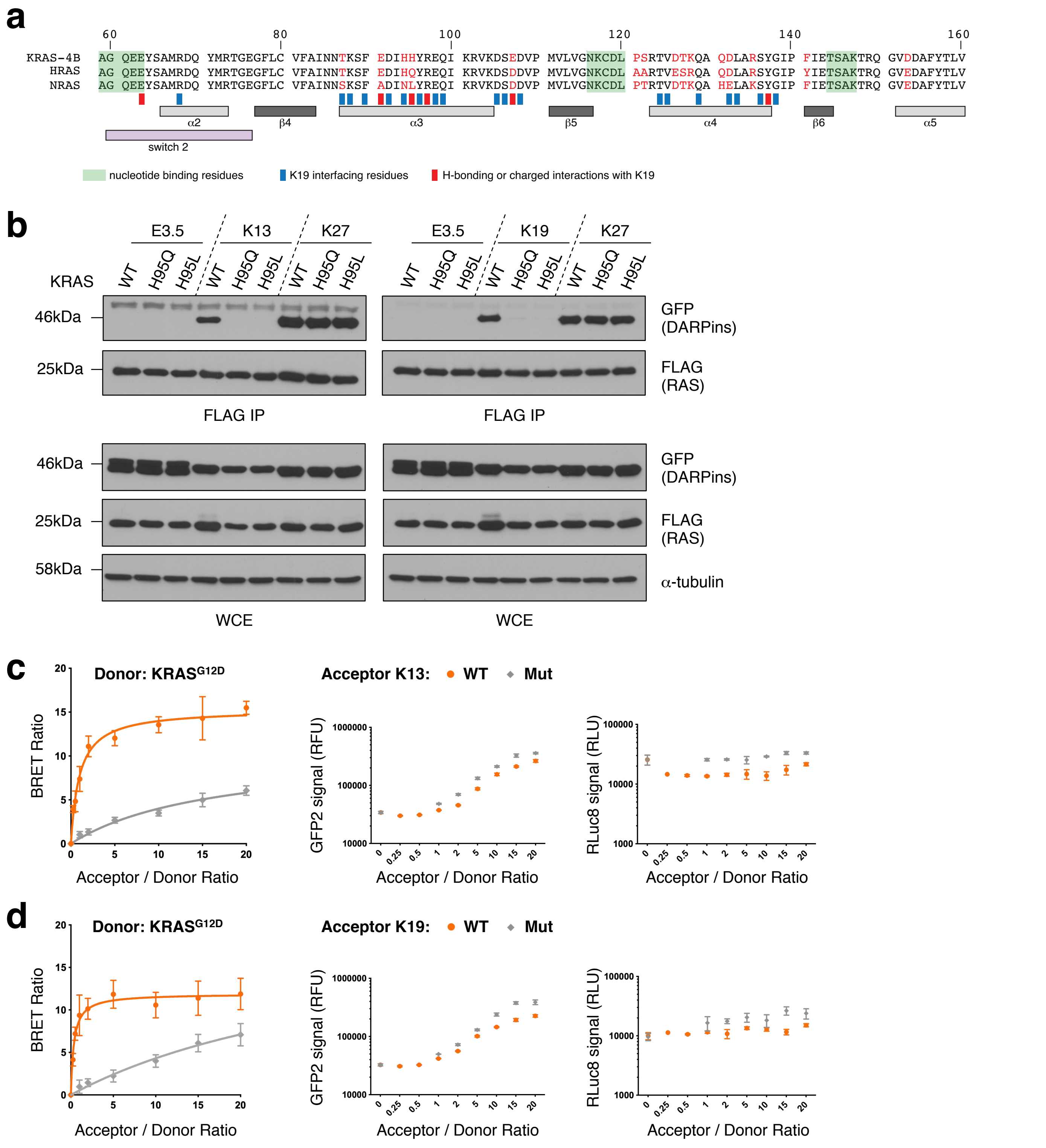

Supplementary Figure 7: Determination of the residues involved in the DARPins selectivity to KRAS. **(a)** Interactions map of KRAS residues in contact with DARPin K19. RAS secondary structures are shown below the amino acid sequence. The loop 7 is between α3 and β5 structures. **(b)** Co-immuno-precipitation of 3xFLAG-$KRAS^{WT}$, -$KRAS^{H95Q}$ and -$KRAS^{H95L}$ with the DARPin-GFP^2 fusions. IP: Immuno-Precipitation, WCE: Whole Cell Extract. **(c-d)** BRET donor saturation assay between $KRAS^{G12D}$ (donor) and K13 mutant (c) or K19 mutant (d) (acceptors) with the GFP^2 and RLuc8 signal controls. Mut corresponds to the substitution of WGW residues (position 35-37) into GGG residues, located in the repeat 1 of the DARPins sequence. Each experiment was performed twice (b-d). Error bars are mean ± SD of biological repeats. **b** Source data are provided as a Source Data file.

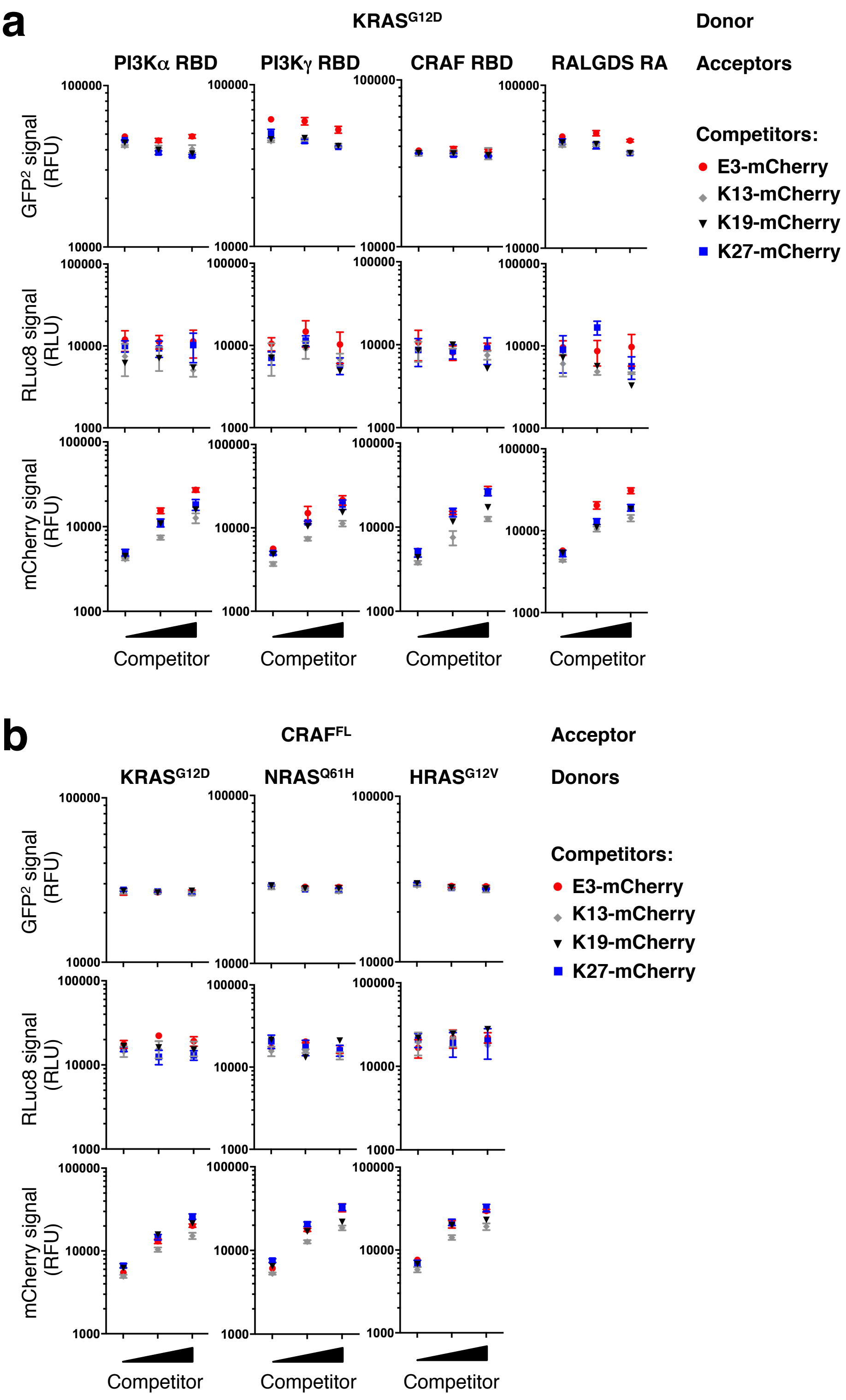

Supplementary Figure 8: K13/K19 selectively prevent KRAS/effector interactions. (a-b) Control of expression of RLuc8 (donor), GFP^2 (acceptors) and mCherry (competitors) signals from the BRET data shown in Figure 4a-d. DARPin E3.5 is a negative and DARPin K27 is a positive control. Each experiment was performed three times (a-b). Error bars are mean ± SD of biological repeats.

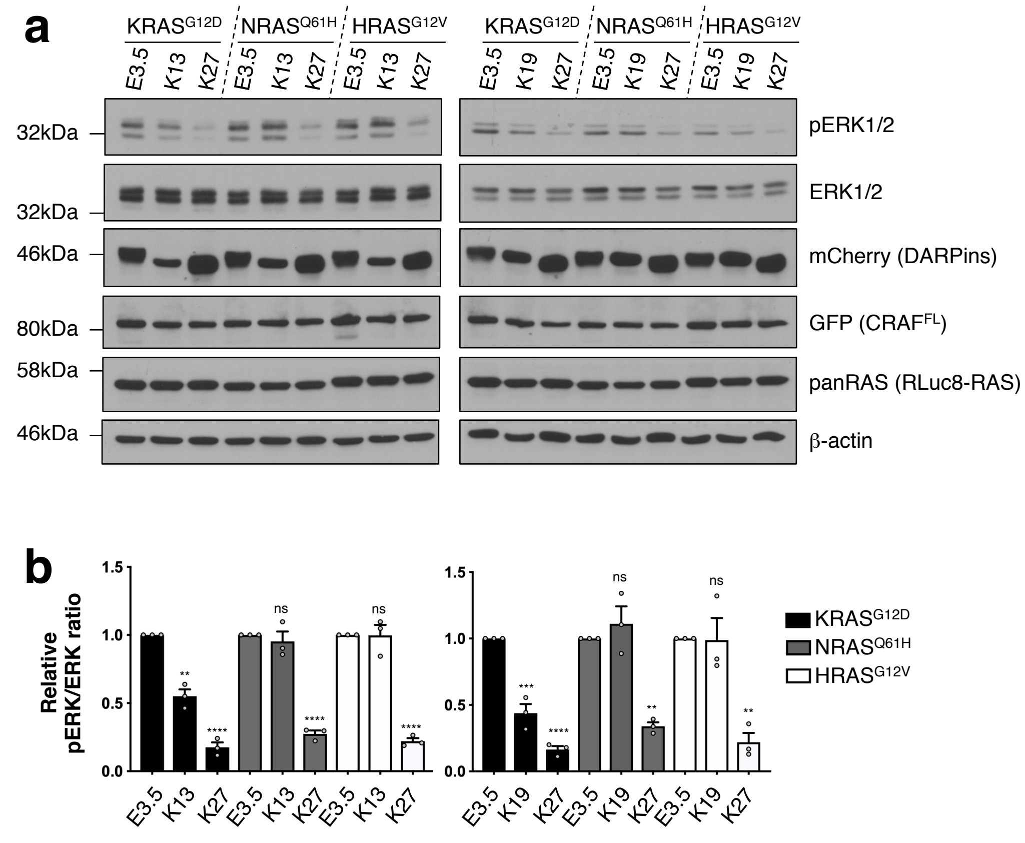

Supplementary Figure 9: Impact of K13/K19 expression on the RAS/RAF/MEK/ERK pathway. (a) Western blot analyses of the activation state of the RAF/MEK/ERK pathway in HEK293T cells overexpressing the DARPin-mCherry fusions with either RLuc8-KRAS^{G12D} or RLuc8-NRAS^{Q61H} or RLuc8-HRAS^{G12V} and GFP^2-CRAF^{FL}. The left hand side panel shows the DARPin K13 with relevant controls and the right hand side panel shows the K19 with the relevant controls. **(b)** Quantification of pERK/ERK signal from (a) was normalized to the negative control E3.5. Statistical analyses were performed using a one-way ANOVA followed by Dunnett's post-hoc tests (**P<0.01, ***P<0.001, ****P<0.0001, ns: not significant). Experiments were performed three times (a, b). Error bars are mean ± SEM of biological repeats. **a** and **b** Source data are provided as a Source Data file.

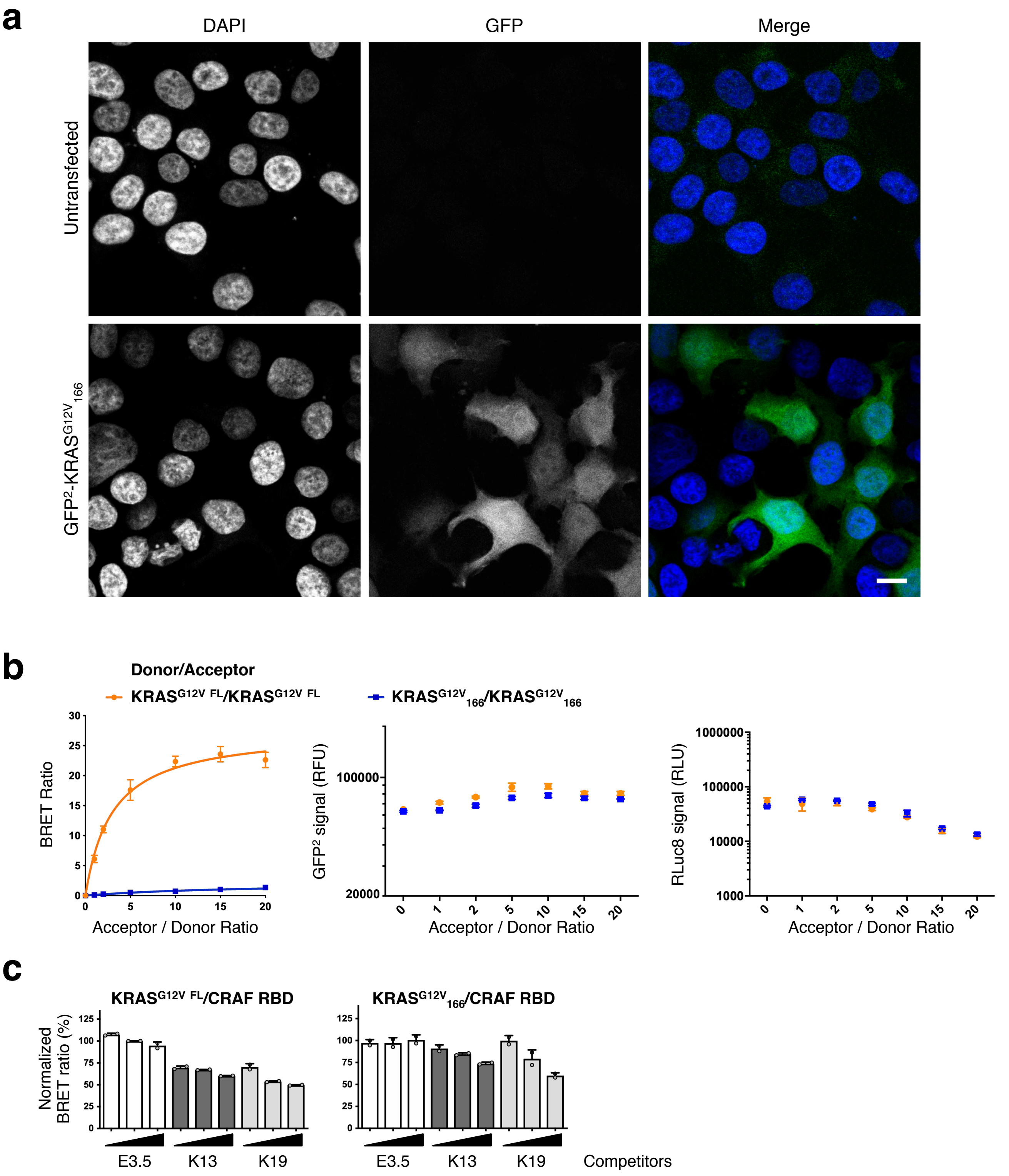

Supplementary Figure 10: K13/K19 inhibit both dimerization and nucleotide exchange of KRAS. (a) Confocal images of untransfected HEK293T cells (top panel) and GFP²-KRAS$^{G12V}_{166}$ transfected HEK293T cells (bottom panel) with DAPI, GFP and both channels merged. Scale bar: 10 μm. **(b)** BRET donor saturation assays showing the dimerization potential of full-length KRAS^{G12V} (as donor and acceptor) and KRAS$^{G12V}_{166}$ (as donor and acceptor). The GFP² and RLuc8 signal controls are also shown. **(c)** BRET competition assays between CRAF RBD/KRAS$^{G12V\ FL}$ and KRAS$^{G12V}_{166}$ interactions with the DARPins as competitors. Each experiment was performed twice (a, c) or three times (b). Error bars are mean ± SD of biological repeats. **c** Source data are provided as a Source Data file.

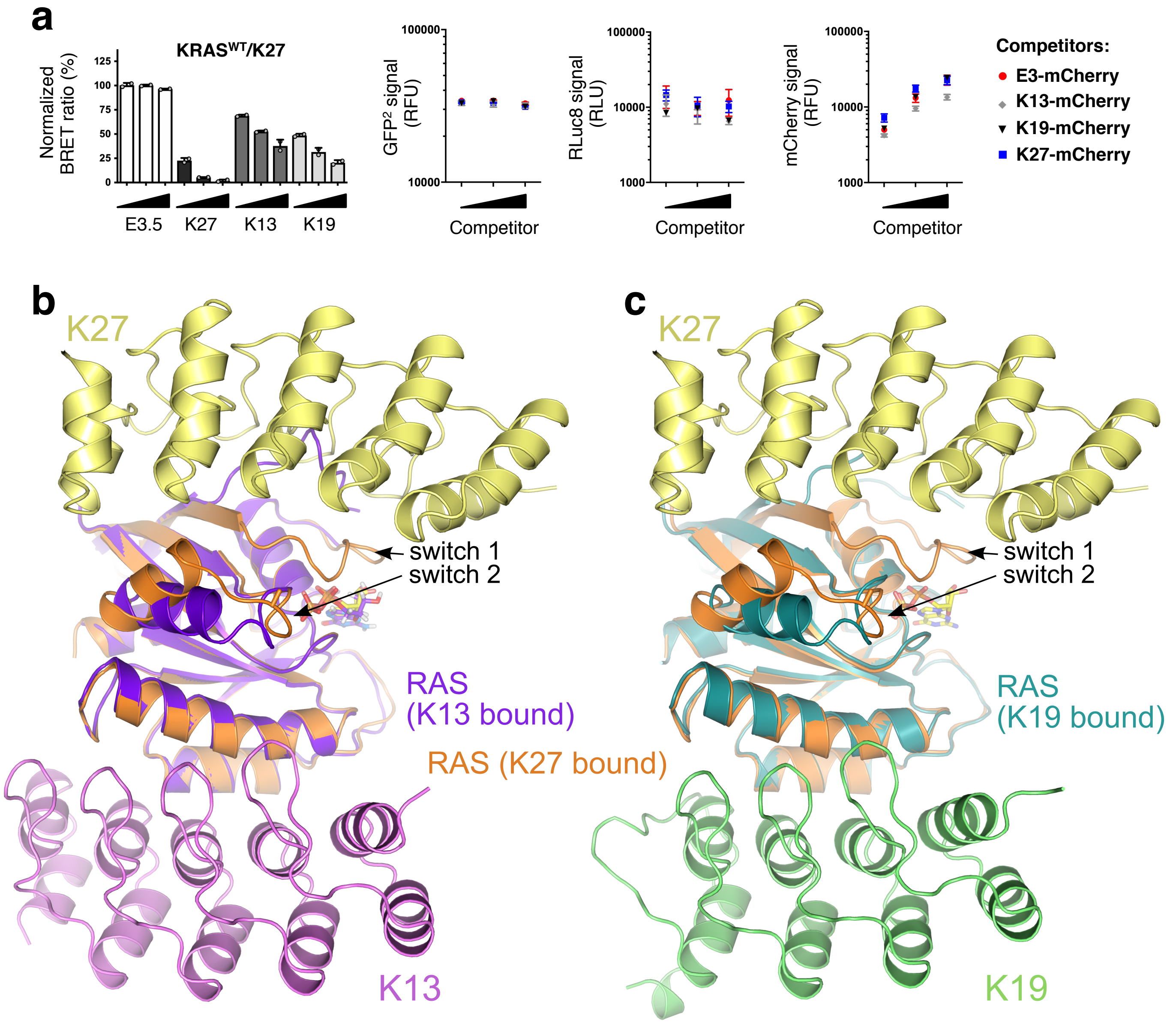

Supplementary Figure 11: K13/K19 inhibit KRAS^{WT}/K27 interaction. **(a)** BRET competition assay of KRAS^{WT}/K27 interaction with the DARPins used as competitor. Control of expression of the acceptor (K27-GFP^2), donor (RLuc8-KRAS^{WT}) and competitors (DARPins-mCherry) are shown. **(b)** Superimposition of KRAS^{G12V}-GDP (purple) bound to K13 (pink, PDB 6H46) and KRAS^{G12V}-GDP (orange) bound to K27 (yellow, PDB 5O2S). **(c)** Superimposition of KRAS^{G12V}-GDP (blue) bound to K19 (green, PDB 6H47) and KRAS^{G12V}-GDP (orange) bound to K27 (yellow, PDB 5O2S). Experiment in a was performed twice. Error bars are mean ± SD of biological repeats. **a** Source data is provided as a Source Data file.

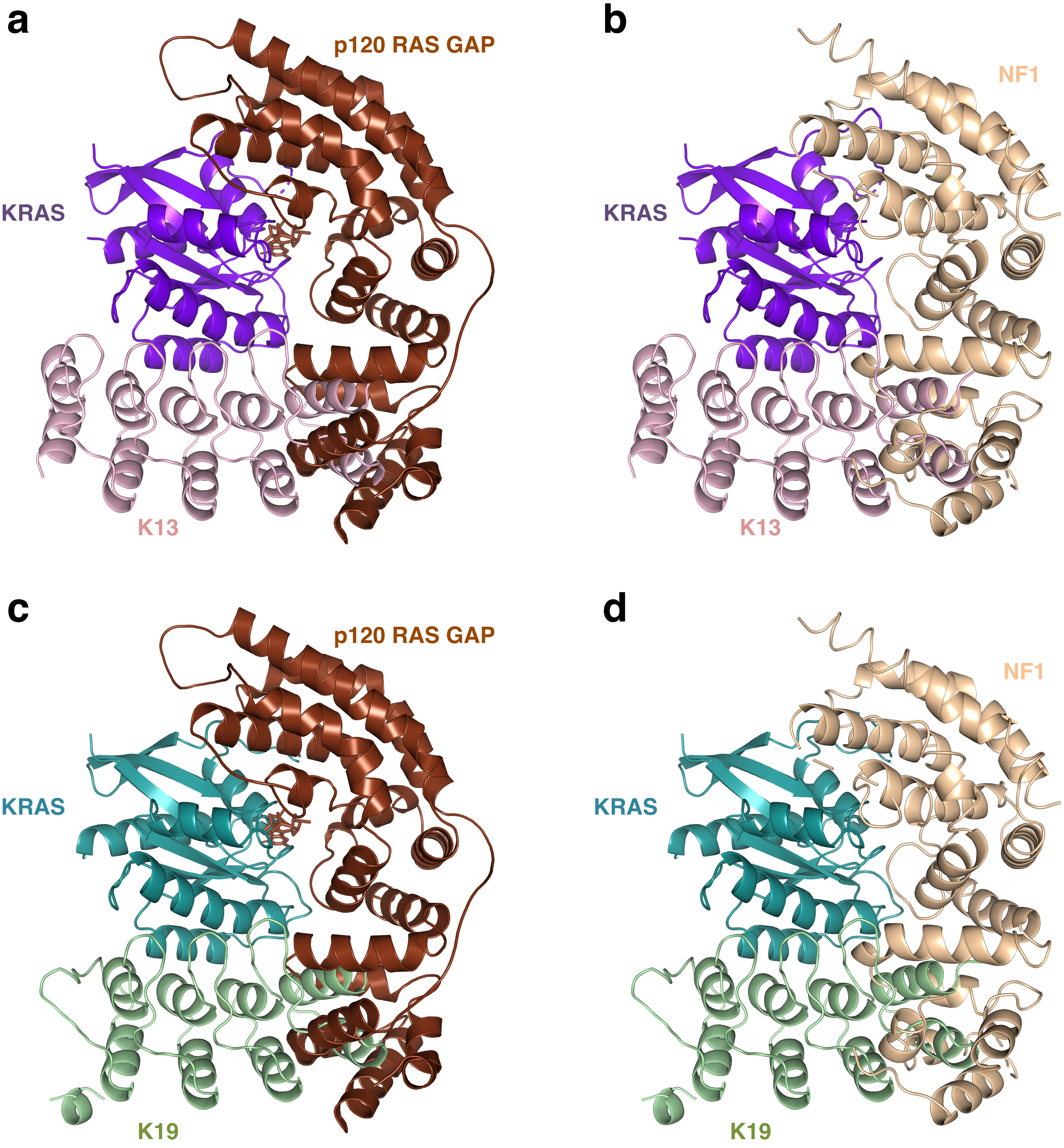

Supplementary Figure 12: K13/K19 interfere with KRAS^{WT}/GAP interactions. (a) Superimposition of KRAS^{G12V}-GDP/K13 structure (KRAS shown in purple and K13 in pink, PDB 6H46) onto the p120-RAS GAP/HRAS^{WT}-GDP structure (only p120-RAS GAP is shown in brown, PDB 1WQ1) or **(b)** onto the NF1 structure (light yellow, PDB 1NF1). **(c)** Superimposition of KRAS^{G12V}-GDP/K19 structure (KRAS shown in blue and K19 in green, PDB 6H47) onto the p120-RAS GAP/HRAS^{WT}-GDP structure (only p120-RAS GAP is shown in brown, PDB 1WQ1) or **(d)** onto the NF1 structure (light yellow, PDB 1NF1).

Supplementary Table 1: Data collection and refinement statistics

	K13	K19
**PDB accession code**	6H46	6H47
**Data collection**		
Space group	$P4_12_12$	$P6_122$
Cell dimensions		
a, *b*, *c* (Å)	79.295, 79.295. 130.025	85.829, 85.829, 227.122
α, β, γ (°)	90, 90, 90	90, 90, 120
Resolution (Å)	2.22-51.5 (2.22-2.28) *	1.70-113.56 (1.70-1.72)
R_{merge}	0.99 (0.68)	0.05 (0.656)
$I / \sigma I$	20.9 (4.0)	30.0 (2.4)
Completeness (%)	99.9 (99.2)	100 (99.3)
Redundancy	13.1 (12.1)	16.5 (7.5)
**Refinement**		
Resolution (Å)	2.22-23.59 (2.22-2.27)	1.70-74.3 (1.70-1.74)
No. reflections	20043 (1423)	52806 (3794)
R_{work} / R_{free}	0.184/0.230	0.192/0.207
No. atoms		
Protein	2520	2364
Ligand/ion	38	20
Water	224	200
B-factors		
Protein	35.1	30.9
Ligand/ion	27.6	50.6
Water	39.7	41.37
R.m.s. deviations		
Bond lengths (Å)	0.0088	0.0068
Bond angles (°)	1.29	1.14

*Values in parentheses are for highest-resolution shell. One crystal was used in both experiments.

Supplementary Table 2: Effect of RAS mutations on the binding of the DARPins based on the BRET and immuno-precipitation data

RAS mutation	DARPins	Binding
$KRAS^{WT}$	K13/K19	+
	K27	+
$KRAS^{H95Q}$	K13/K19	-
	K27	+
$KRAS^{H95L}$	K13/K19	-
	K27	+
$KRAS^{E107D}$	K13/K19	+
	K27	+
$KRAS^{G12D}$	K13/K19	+
	K27	+
$KRAS^{S17N}$	K13/K19	+
	K27	-
$NRAS^{WT}$	K13/K19	-
	K27	+
$NRAS^{Q61H}$	K13/K19	-
	K27	+
$HRAS^{WT}$	K13/K19	-
	K27	+
$HRAS^{G12V}$	K13/K19	-
	K27	+

Supplementary Table 3: Primers used in this study

Primers name	Primers sequence (5' - 3')
KRASNotI For	TAAATTGCGGCCGCTATGACCGAATATAAACTTGTGGTAG
$KRAS_{166}$XbaI Rev	GAATAATCTAGATTAATGTTTTCGAATTTCTCG
$KRAS^{FL}$XbaI Rev	GAATAATCTAGATTACATAATTACACACTTTGTC
KRASH95Q For	GAAGATATTCACCAGTATAGAGAAC
KRASH95Q Rev	GTTCTCTATACTGGTGAATATCTTC
KRASH95L For	GAAGATATTCACCTCTATAGAGAAC
KRASH95L Rev	GTTCTCTATAGAGGTGAATATCTTC
KRASE107D For	GAGTTAAGGACTCTGATGATGTACCTATGG
KRASE107D Rev	CCATAGGTACATCATCAGAGTCCTTAACTC
NRASWT For	GATACAGCTGGACAAGAAGAGTACAG
NRASWT Rev	CTGTACTCTTCTTGTCCAGCTGTATC
HRASWT For	TTGTTGGCGCCGGCGGTGTGGGCAA
HRASWT Rev	TTGCCCACACCGCCGGCGCCAACAA
HRASNotI For	TAAATTGCGGCCGCTATGACCGAATACAAGCTTGTTG
HRASXbaI For	GAATAATCTAGATCAGGAGAGCACACACTTGC
NRASNotI For	TAAATTGCGGCCGCTATGACTGAGTACAAACTGG
NRASXbaI Rev	GAATAATCTAGATTACATCACCACACATGGC
DARPinNcoI For	ATTATTCCATGGATCTGGGAAAAAAACTGCTGG
DARPinXhoI Rev	AATAATCTCGAGCAGTTTCTGCAGGATTTCC
DARPinE3.5XhoI Rev	AATAATCTCGAGCTGCAGGATCTCGGCCAGG
DARPin-RD1	AGACCACAACGGTTTCCCTCTAGAAATAATTTTGTTTAACT<br>TTAAGAAGGAGATATATCCATGGCCGATCTGGGAAA
pET16b_DP_For	CGATCATATGGATCTGGGAAAAAAACTGCTGGA
pET16b_DP_Rev	ATCGGGATCCTCACTACAGTTTCTGCAGG
