# Supplementary material for: KRAS-specific inhibition using a DARPin binding to a site in the allosteric lobe
Source: Nat Commun. 2019 Jun 13;10:2607. doi: 10.1038/s41467-019-10419-2 (PMC6565726; doi:10.1038/s41467-019-10419-2)
Supplement: Supplementary file 2 — Reporting Summary [file 41467_2019_10419_MOESM2_ESM.pdf]

nature research

Corresponding author(s): T.H. Rabbitts

Reporting Summary

Nature Research wishes to improve the reproducibility of the work that we publish. This form provides structure for consistency and transparency in reporting. For further information on Nature Research policies, see Authors & Referees and the Editorial Policy Checklist.

Statistical parameters

When statistical analyses are reported, confirm that the following items are present in the relevant location (e.g. figure legend, table legend, main text, or Methods section).

n/a	Confirmed	
☐	☒	The exact sample size (n) for each experimental group/condition, given as a discrete number and unit of measurement
☐	☒	An indication of whether measurements were taken from distinct samples or whether the same sample was measured repeatedly
☐	☒	The statistical test(s) used AND whether they are one- or two-sided<br>*Only common tests should be described solely by name; describe more complex techniques in the Methods section.*
☒	☐	A description of all covariates tested
☒	☐	A description of any assumptions or corrections, such as tests of normality and adjustment for multiple comparisons
☐	☒	A full description of the statistics including central tendency (e.g. means) or other basic estimates (e.g. regression coefficient) AND variation (e.g. standard deviation) or associated estimates of uncertainty (e.g. confidence intervals)
☒	☐	For null hypothesis testing, the test statistic (e.g. F, t, r) with confidence intervals, effect sizes, degrees of freedom and P value noted<br>*Give P values as exact values whenever suitable.*
☒	☐	For Bayesian analysis, information on the choice of priors and Markov chain Monte Carlo settings
☒	☐	For hierarchical and complex designs, identification of the appropriate level for tests and full reporting of outcomes
☒	☐	Estimates of effect sizes (e.g. Cohen's d, Pearson's r), indicating how they were calculated
☐	☒	Clearly defined error bars<br>*State explicitly what error bars represent (e.g. SD, SE, CI)*

Our web collection on statistics for biologists may be useful.

Software and code

Policy information about availability of computer code

Data collection	*Provide a description of all commercial, open source and custom code used to collect the data in this study, specifying the version used OR state that no software was used.*
Data analysis	All data analysis were performed using ImageJ or Prism 7.0 c (GraphPad Software)

For manuscripts utilizing custom algorithms or software that are central to the research but not yet described in published literature, software must be made available to editors/reviewers upon request. We strongly encourage code deposition in a community repository (e.g. GitHub). See the Nature Research guidelines for submitting code & software for further information.

Data

Policy information about availability of data

All manuscripts must include a data availability statement. This statement should provide the following information, where applicable:

- Accession codes, unique identifiers, or web links for publicly available datasets
- A list of figures that have associated raw data
- A description of any restrictions on data availability

Structure files and coordinates have been deposited to PDB under these accession numbers: 6H46 and 6H47.

Field-specific reporting

Please select the best fit for your research. If you are not sure, read the appropriate sections before making your selection.

☒ Life sciences ☐ Behavioural & social sciences ☐ Ecological, evolutionary & environmental sciences

For a reference copy of the document with all sections, see nature.com/authors/policies/ReportingSummary-flat.pdf

Life sciences study design

All studies must disclose on these points even when the disclosure is negative.

Sample size	*Describe how sample size was determined, detailing any statistical methods used to predetermine sample size OR if no sample-size calculation was performed, describe how sample sizes were chosen and provide a rationale for why these sample sizes are sufficient.*
Data exclusions	*Describe any data exclusions. If no data were excluded from the analyses, state so OR if data were excluded, describe the exclusions and the rationale behind them, indicating whether exclusion criteria were pre-established.*
Replication	*Describe the measures taken to verify the reproducibility of the experimental findings. If all attempts at replication were successful, confirm this OR if there are any findings that were not replicated or cannot be reproduced, note this and describe why.*
Randomization	*Describe how samples/organisms/participants were allocated into experimental groups. If allocation was not random, describe how covariates were controlled OR if this is not relevant to your study, explain why.*
Blinding	*Describe whether the investigators were blinded to group allocation during data collection and/or analysis. If blinding was not possible, describe why OR explain why blinding was not relevant to your study.*

Reporting for specific materials, systems and methods

Materials & experimental systems

n/a	Involved in the study	
☒	☐	Unique biological materials
☐	☒	Antibodies
☐	☒	Eukaryotic cell lines
☒	☐	Palaeontology
☒	☐	Animals and other organisms
☒	☐	Human research participants

Methods

n/a	Involved in the study	
☒	☐	ChIP-seq
☒	☐	Flow cytometry
☒	☐	MRI-based neuroimaging

Antibodies

Antibodies used

Phospho-ERK 1/2, Rabbit antibody, Cell Signaling Technology, Cat#9101S RRID:AB_331646
Total ERK 1/2, Rabbit antibody, Cell Signaling Technology, Cat#9102S RRID:AB_330744
Phospho-MEK 1/2, Rabbit antibody, Cell Signaling Technology, Cat#9154S RRID:AB_2138017
Total MEK 1/2, Mouse antibody, Cell Signaling Technology, Cat#4694S RRID:AB_10695868
Phospho-AKT S473, Rabbit antibody, Cell Signaling Technology, Cat#4058S RRID:AB_331168
Phospho-AKT T308, Rabbit antibody, Cell Signaling Technology, Cat#4056S RRID:AB_331163
Total AKT, Rabbit antibody, Cell Signaling Technology, Cat#9272S RRID:AB_329827
GFP, Mouse antibody, Santa Cruz Biotechnology, Cat#sc-9996 RRID:AB_627695
β-Actin, Mouse antibody, Sigma-Aldrich, Cat#A1978 RRID:AB_476692
DsRed (cross react with mCherry), Goat antibody, Santa Cruz Biotechnology, Cat#sc-33353 RRID:AB_639924
Pan-RAS, Mouse antibody, Millipore, Cat#OP40 RRID:AB_213400
α–tubulin, Rabbit antibody, Abcam, Cat#ab4074 RRID:AB_228800
FLAG tag, Mouse antibody, Sigma, Cat#F3165 RRID:AB_259529

Validation

All these antibodies have been validated for western blot use (see manufacturers website).

Eukaryotic cell lines

Policy information about cell lines

Cell line source(s)	HEK293T, ATCC, Cat#CRL-3216 RRID:CVCL_0063<br>HCT116, ATCC, Cat# CCL-247, RRID:CVCL_0291<br>MCF-7, ATCC, Cat# HTB-22, RRID:CVCL_0031<br>HT1080, ATCC, Cat# CCL-121, RRID:CVCL_0317<br>T24, ATCC, Cat# HTB-4, RRID:CVCL_0554
Authentication	Mutation detection of RAS mutations using RT-PCR for the cancer cell lines
Mycoplasma contamination	All cell lines tested negative for mycoplasma contamination
Commonly misidentified lines<br>(See ICLAC register)	*Name any commonly misidentified cell lines used in the study and provide a rationale for their use.*
